# Spontaneous eye movements during eyes-open rest reduce resting-state-network modularity by increasing visual-sensorimotor connectivity

**DOI:** 10.1162/netn_a_00186

**Published:** 2021-06-03

**Authors:** Cemal Koba, Giuseppe Notaro, Sandra Tamm, Gustav Nilsonne, Uri Hasson

**Affiliations:** MoMiLab Research Unit, IMT School for Advanced Studies Lucca, Lucca, Italy; Center for Mind/Brain Sciences (CIMeC), The University of Trento, Trento, Italy; Department of Clinical Neuroscience, Karolinska Institutet, Solna, Sweden; Department of Psychology, Stockholm University, Stockholm, Sweden; Department of Psychiatry, Oxford University, Oxford, UK; Department of Clinical Neuroscience, Karolinska Institutet, Solna, Sweden; Department of Psychology, Stockholm University, Stockholm, Sweden; Center for Mind/Brain Sciences (CIMeC), The University of Trento, Trento, Italy

**Keywords:** Eye-movements, Resting-state, Networks, Modularity, Eye-orbit

## Abstract

During wakeful rest, individuals make small eye movements during fixation. We examined how these endogenously driven oculomotor patterns impact topography and topology of functional brain networks. We used a dataset consisting of eyes-open resting-state (RS) fMRI data with simultaneous eye tracking. The eye-tracking data indicated minor movements during rest, which correlated modestly with RS BOLD data. However, eye-tracking data correlated well with echo-planar imaging time series sampled from the area of the eye-orbit (EO-EPI), which is a signal previously used to identify eye movements during exogenous saccades and movie viewing. Further analyses showed that EO-EPI data were correlated with activity in an extensive motor and sensorimotor network, including components of the dorsal attention network and the frontal eye fields. Partialling out variance related to EO-EPI from RS data reduced connectivity, primarily between sensorimotor and visual areas. It also produced networks with higher modularity, lower mean connectivity strength, and lower mean clustering coefficient. Our results highlight new aspects of endogenous eye movement control during wakeful rest. They show that oculomotor-related contributions form an important component of RS network topology, and that those should be considered in interpreting differences in network structure between populations or as a function of different experimental conditions.

## Author Summary

We studied how subtle eye movements made during fixation, in absence of any other task, are related to resting-state connectivity measured using fMRI. We used a dataset for which eye tracking and BOLD resting-state were acquired simultaneously. We correlated brain activity with both eye-tracking metrics as well as time series sampled from the area of the eye orbits (EO-EPI). Eye-tracking data correlated well with the EO-EPI data. Furthermore, EO-EPI correlated with BOLD signal in sensorimotor and visual brain systems. Removing variance related to EO-EPI reduced connectivity between sensorimotor and visual areas and resulted in more modular resting-state networks. Our findings show that oculomotor-related contributions are an important component of resting-state network topology, and that they can be studied using EPI data from the eye orbits.

## INTRODUCTION

The study of human brain activity during resting state (RS) is of considerable interest in both basic and clinical brain research. For mechanistically oriented perspectives, RS activity patterns identify constraints that may govern task-evoked activity as seen by relations between RS connectivity and interindividual differences in various cognitive tasks (e.g., Kelly, Uddin, Biswal, Castellanos, & Milham, 2008; Rosenberg, Hsu, Scheinost, Constable, & Chun, 2018). And because RS connectivity is related to structural connectivity (e.g., Honey et al., 2009; Mišic´ et al., 2016), it is considered an important mediator between anatomical organization and task-evoked activity. From the perspective of predictive models of interindividual differences in healthy and clinical populations, the quantification of RS features (using time-domain, network-based analyses, spatiotemporal clustering, or control-based approaches, to name a few) is used for machine learning or statistical learning. This has proved promising in contexts such as prediction of IQ (e.g., Dubois, Galdi, Paul, & Adolphs, 2018), personality (e.g., Nostro et al., 2018), or the likelihood of developing clinical conditions (e.g., de Vos et al., 2018).

Resting-state data measured via fMRI reflect endogenous neural activity, but also additional sources that introduce fluctuations in the signal. Some of these are physiological artifacts (e.g., cardiac and respiratory effects, Birn, 2012; J. Chen et al., 2020), or head and body motion (e.g., Parkes, Fulcher, Yücel, & Fornito, 2018). For machine learning, these nonneural effects on the BOLD signal may be informative; for example, motion-related patterns could differ across populations (e.g., Zacà, Hasson, Minati, & Jovicich, 2018). However, motion and physiological effects complicate drawing conclusions about brain systems mediating endogenous information-computation during wakeful rest. For this reason, researchers often remove effects of motion and physiology from RS data prior to analysis, even though some effects of physiology could be meaningfully related to central neural systems involved in control of autonomic activity (e.g., Iacovella, Faes, & Hasson, 2018; Iacovella & Hasson, 2011).

Here we examined how RS connectivity is related to a distinct factor, which is eye movement during rest (while fixating with eyes open). For purposes of understanding endogenous computations, spontaneous eye movement at rest straddles the boundary between an interesting neurobiological phenomenon reflecting the output of endogenous activity and a nuisance factor reflecting motor activity. On one hand, eye movement can be considered a truly integral component of wakeful rest, because at minimum, retinal input is continuously refreshed to minimize adaptation (for review, see Rucci & Poletti, 2015). On the other hand, oculomotor control differs from prototypical covert, nonmotor processes exactly because motor control involves planning, execution, efference copy, feedback, and correction (e.g., West, Welsh, & Pratt, 2009). Oculomotor control during rest may therefore require coordination between brain systems that otherwise present modest levels of connectivity.

Statistically, eye movements during rest could therefore produce stronger connectivity between regions. Perhaps more importantly, it could produce a more integrated (less-modular) view of RS connectivity networks, because eye movement is supported by a widely distributed fronto-parietal network and occipital regions (e.g., Balslev, Albert, & Miall, 2011; Mort et al., 2003). From a theoretical perspective, identifying neural systems controlling eye movement during rest could allow better partitioning between relatively more ‘active,’ (oculo)motor-related aspect of RS as opposed to other more covert, nonmotor-related aspects of RS. Finally, eye movements themselves could be a possible confounder when studying healthy and clinical populations that differ in oculomotor control, including autism (e.g., Takarae, Minshew, Luna, Krisky, & Sweeney, 2004), Parkinson’s disease (e.g., Pretegiani & Optican, 2017; Zhang et al., 2018), or schizophrenia (e.g., Dowiasch et al., 2016; Morita, Miura, Kasai, & Hashimoto, 2020).

### Current Knowledge

There is relatively little prior work on the relationship between eye movements and RS activity. Using fMRI, Fransson, Flodin, Seimyr, and Pansell (2014) studied neural correlates of horizontal or vertical guided fixations, as well as spontaneous fixations during RS. Guided fixations produced activity in systems typically involved in oculomotor movement, including visual cortex, frontal eye fields (FEF), supplementary motor area (SMA), cerebellum, and a few other regions. To quantify correlates of spontaneous eye movement during RS, they derived a gaze-velocity time series from the eye tracking data, reduced its dimensionality using PCA, convolved the resulting time series with a hemodynamic response function, and used the result as a regressor in a whole-brain analysis. Interestingly, this latter analysis identified fewer regions, which furthermore did not overlap with those found for guided saccades, and which were all associated with the default mode network (DMN): the posterior cingulate cortex (PCC) and dorsomedial prefrontal cortex (dmPFC). As the authors noted, “at first glance it would seem more likely to expect the neuronal control for slow changes in eye position during fixation to be localized to visual cortices and attention-related cortical networks” (Fransson et al., 2014, p. 3833). It is unclear how slow fluctuations in the DMN impact eye movement.

McAvoy et al. (2012) used electrooculography (EOG) to monitor eye movement during fixation, in an analysis based on a relatively small sample of nine participants. Using EOG, they separated blinks from other eye movement during eyes-open RS. In the analysis of EOG during RS fixation they identified brain systems correlated with blinks, but no brain systems where activity correlated with other types of eye movements.

Yellin, Berkovich-Ohana, and Malach (2015) examined correlations between fMRI BOLD fluctuations during rest and pupil size. They identified widespread negative correlations in sensorimotor areas and temporal areas, and positive correlations in the DMN. The study did not evaluate BOLD correlates of gaze location or velocity. However, it is possibly related to understanding systems related to spontaneous eye movement, because pupil size measurements are known to be confounded with the deviation of the pupil from the center of camera view. That is, eye trackers will misreport systematically decreasing pupil size values—for the exact same pupil size—as the pupil deviates from the camera axis (Hayes & Petrov, 2016). This mismeasurement is known as the pupil foreshortening error (PFE). Specifically, Hayes and Petrov (2016) showed that deviations from center of camera view produce systematic PFEs that can reach 12% at typical viewing distances. Significant PFEs were produced even with movements as small as 4° from center.

Ramot et al. (2011) used EOG to determine BOLD correlates of spontaneous eye movements during an eyes-closed condition. The relation to eyes-open oculomotor control is unclear, as eyes-closed RS conditions produce different patterns of brain activity (e.g., Marx et al., 2003) and connectivity (e.g., McAvoy et al., 2012). Furthermore, saccades made under closed eye lids have different trajectories than those made with eyes open in complete darkness (Becker & Fuchs, 1969). For this reason we consider prior studies examining RS activity during eyes-open condition as more relevant for the current study.

In addition, numerous neuroimaging studies have used various types of tasks, including visually-guided saccades, memory-guided saccades, antisaccades, and so-called “voluntary” saccades (either precued [endogenous control] or freely initiated). However, these studies used explicit tasks rather than study naturally occurring oculomotor control during eyes-open RS. Perhaps the essential difference is that controlled studies oftentimes orient the saccade toward or away from a presented target (pro- vs. antisaccade). For this reason the brain systems identified could mediate visual detection and attention processes that have no parallel during rest. In a neuroimaging study demonstrating this point (Brown, Goltz, Vilis, Ford, & Everling, 2006), participants were required to saccade either toward a stimulus (prosaccade), away from a stimulus (antisaccade), or maintain fixation while inhibiting an orienting saccade (no-go). They documented numerous regions, including FEF, IPS, cingulate cortex, and precuneus, all showing highly similar activation patterns for both prosaccade and no-go trials. The authors interpreted this as suggesting that “BOLD signal in cortical saccade regions might predominantly reflect visual detection and attention processes rather than saccade generation or inhibition…” For this reason, it is unclear to what extent brain systems identified in typical studies of saccades are strongly involved in saccade control during the resting state.

### Specific Aims

The two aims of our current study were: (a) to identify brain systems associated with endogenously driven eye movements during rest, and conjointly, (b) to determine how removal of eye movement–related activity impacts resting-state connectivity. We quantified eye movement during rest using both eye tracking and EPI data extracted from the eye orbit area. We validated the relationship between different features of eye movement (pupil size, gaze velocity, gaze location) and eye orbit EPI time series (EO-EPI) during rest. We then evaluated how removal of eye-related activity, as manifested in EO-EPI, impacts the topography and topology of RS networks. In doing so, we examine how EO-EPI removal impacts global metrics of network connectivity including modularity, number of modules, and properties of the degree distribution because these speak to large-scale, holistic changes to brain networks. In addition, we quantify the impact of EO-EPI removal on other, local metrics of connectivity (e.g., mean degree) in order to allow relating past and future results to our results.

## METHODS

### Dataset

We used resting-state data from the Sleepy Brain study (Nilsonne et al., 2016). All data are available online from OpenNeuro, Dataset ds000201 (https://www.openneuro.org/datasets/ds000201/). Full details of the dataset and imaging parameters are given in Nilsonne et al. (2016), and here we provide only the main details. Data were collected from 86 participants on a 3T MRI scanner (Discovery 750, General Electric) using an 8-channel head coil. Each participant was scanned on two different days. In each scanning session, a T1 structural image, two resting-state functional EPI scans, and three task-related functional scans (emotional mimicry, empathy for pain, emotional reappraisal) were acquired. Our analyses rely only on the structural and resting-state scans.

For the structural (T1) images, the relevant properties were as follows: slice thickness 1 mm, sagittal orientation, whole-brain acquisition; for the resting state EPI images: slice thickness 3 mm no gap, axial orientation, 49 slices covering the entire brain, interleaved acquisition inferior to superior, *TE* = 30, *TR* = 2.5 sec, flip angle 75°.

Four resting-state datasets were acquired for each participant; two runs on each of two scanning days. In one of the two days, data were collected when participants were sleep deprived, and we did not analyze these data. Of the remaining two RS runs, one was typical, where participants were asked to fixate on a white cross presented a gray background for 8 minutes. The second run was quasirest in that in addition to fixation, it included self-rated sleepiness probes every 2 minutes. We only analyzed data from the typical RS session. To summarize, we processed one RS run per participant, which was a typical RS scan acquired in absence of sleep deprivation. Three participants did not provide these runs so 83 participants were included in our initial sample. Participants belonged to two age groups: 20–30 years of age (*n* = 45, *Median* = 23) and 65–75 years of age (*n* = 38, *Median* = 68). We did not have specific hypotheses about how age may mediate correlations between eye movement and BOLD. Therefore, in investigating potential age effects, our main intention was to understand whether this factor confounded any of the reported analyses. Because of the large difference between the age distributions, we treated age as a categorical variable (age group) rather than as a continuous one.

### Pre-Processing of Eye Tracking Data

Eye tracking data were available for 77 of the 83 participants for which we analyzed the RS data. Participants were required to maintain their gaze on a central fixation cross for the duration of the 8-min scan. Right-eye movement and pupil size were recorded using a ViewPoint EyeTracker (Arrington Research, USA) integrated into head-mounted goggles. Eye data were sampled at 60 Hz. Participants were monitored during the experiment to ensure that they did not have prolonged eye closures (>5 sec).

When analyzing these data we observed a substantial proportion of missing values, likely due to loss of pupil tracking during the task. We therefore implemented a quality assurance procedure as detailed below. We constructed a histogram of the standard deviations of the gaze norm (defined as $\sqrt{{\text{gaze}}_{x}^{2}+{\text{gaze}}_{y}^{2}}$); see Supporting Information Figure S1. On the basis of the distribution of these values and visual inspection of the data, we set the upper bound to *SD*_*gaze*_ = 0.32 and excluded participants with *SD*_*gaze*_ above this threshold. We chose this threshold in order to maintain time series with relatively low proportion of potential artifact peaks, because the adaptive threshold algorithm we use for peak detection (described below) is applicable if peaks are relatively rare as compared to baseline. This step resulted in exclusion of 43 of the 77 datasets.

Manufacturer guidelines define artifacts as measurements where one of the pupil dimensions is outside the range of 0.1–0.5. Based on this definition, we removed an additional two participants for whom more than 50% of measurements were outside this range. For the remaining 32 subjects we performed the following analysis to detect eye blinks and nonblink artifacts, based on estimations of the artifact duration. We first defined an artifact function as the sum of the following three functions (Equations 1–3, each normalized to its maximum value). In these equations, *f*_1_ is the pupil aspect ratio, whereas *f*_2_ and *f*_3_ diverge when one pupil dimension approach the boundaries of the validity range 0.1–0.5.

$$f_{1}=\text{pupilwidth}/\text{pupilheight}$$ (1)

$$f_{2}=1/\left({\text{pupilwidth}}^{2}\right)+1/\left({\text{pupilheight}}^{2}\right)$$ (2)

$$f_{3}=1/\left({\left(\text{pupilwidth}-.6\right)}^{2}\right)+1/\left({\left(\text{pupilheight}-.6\right)}^{2}\right)$$ (3)

To individuate the artifacts’ start and end points, we applied an adaptive algorithm proposed by Nyström and Holmqvist (2010). This algorithm was originally developed for saccade detection using gaze speed as input, and we adapted it to use the absolute value of the artifact function as input. In brief, this method consists of first detecting the peaks of the input through a locally adaptive threshold, which is then followed by detecting the artifact onset and offset as the closest point of minimum below that threshold. Supporting Information Figure S2 shows an example of detected peaks of the artifact function. These peaks correspond to intervals of pupil size measurements outside the validity range.

In summary, we analyzed data from 32 (of 77) participants (25 from the younger participants group, 7 from the older). For these, the proportion of artifacts was on average 18 ± 2%. Blinks occurred with an average period of 2.36 ± 0.21 sec.

### Pre-Processing of fMRI Data and Creation of Eye Orbit EPI Regressors

We include the analysis workflow described below as Supporting Information, also available online via a GitHub repository (Koba, 2021).

First, we applied brain extraction and tissue segmentation (gray matter, white matter, CSF) to the structural T1 images using the *antsBrainExtraction* function of ANTs software (Avants, Tustison, & Song, 2011). We used ANTs for all registration routines in our pipeline. We registered each participant’s structural image to standard space using non-linear registration (ICBM 2009 nonlinear asymetric template; Fonov, Evans, McKinstry, Almli, & Collins, 2009) and saved the inverse of the warps. We also registered the structural and functional images using affine transformation. We used the combination of these two transformations to align data from each participant’s original space to common space, or vice versa, in a single step.

To delineate each participants “eye orbit” area, we first marked this area on the common space template. We then transformed this mask to each participant’s original space and made any additional modifications therein, if required. Specifically, we delineated anatomical masks of the “eye orbit” area in common space using MRICRON (Rorden, Karnath, & Bonilha, 2007), for which we used an MNI template provided with FSL (Jenkinson, Beckmann, Behrens, Woolrich, & Smith, 2012). Both eye orbits were included in the mask. The masks’ location was transformed to each participant’s individual space using the combination of the MNI→T1 and T1→subject space alignment matrices mentioned above. We also created cerebral spinal fluid (CSF) and white matter masks in MNI space and transformed them to individual space, where they were eroded by one voxel from their outer boundaries to be more conservative. We then extracted the mean time series from these white matter and CSF masks. These were used as nuisance regressors in an initial regression (details below).

We used AFNI (Cox, 1996) for analyzing the functional RS images. We implemented the following steps: slice time correction, motion correction (base image: first volume of the run), and band-pass filtering (0.01–0.1 Hz). To remove other nuisance sources of variance from the functional times series, we implemented preliminary data-cleaning using regression with the following regressors: (a) motion parameters estimated during motion correction, (b) mean white matter and CSF time series, and (c) frame-wise displacement values. We considered the residuals of this regression as a “cleaned” time series that was the entry point for further analyses.

To improve signal to noise of the subsequent regression models that were of primary interest, we then spatially smoothed the cleaned time series with a 6-mm FWHM kernel. From this time series we also derived an Eye-Orbit EPI regressor, which was defined as the mean time series from both eye orbit masks, after spatial smoothing, which we refer to as EYE_*raw*_. We convolved the EYE_*raw*_ with an HRF basis function (using AFNI’s *waver* command), producing an EYE_*conv*_ time series. In separate analyses we used either EYE_*raw*_ or EYE_*conv*_ as “seed” regressors to identify EO-EPI-correlated brain areas.

### Determining Correlation Between Eye-Tracking Measures and EO-EPI Time Series

We were interested in the relationship between several measures of eye movement and the EPI time series sampled from the eye orbit regions (EO-EPI series). We derived 12 time series from the eye-tracking data: the measured gaze location, *GazeX* and *GazeY* (mean normalized for horizontal center per participant), their squared values, their temporal derivatives (*vel_GazeX*, *vel_GazeY*), gaze amplitude: *GazeX*^2^ + *GazeY*^2^, gaze power: *vel_GazeX*^2^ + *vel_GazeY*^2^, *Pupil_size* (de-meaned), its first derivative *vel_Pupil_size*, and squared value *Pupil_size*^2^. We were also interested in the *blink function* (coding for 1 whenever a blink was present; 0 otherwise), but we determined the relation between blinks and EO-EPI in a different manner as detailed below. *Pupil_size* was defined as (*pupil_width* + *pupil_height*)/2. We note that with our instrumentation, as well as many other eye trackers, the pupil size measure may be confounded with gaze position (Hayes & Petrov, 2016), resulting in significant correlations between *pupil_size* and gaze location in both *x* and *y* directions (*p* < .01 for 30 of the 32 participants in the current study).

For each of the 12 eye-tracking quantities mentioned above (with the exception of blinks), we performed the following procedure: we first downsampled the time series to the fMRI frequency rate (0.4 Hz). Rather than assume that the relation between the eye tracker data and EO-EPI is mediated by a typical hemodynamic response function, we used a simple statistical learning approach to estimate and validate this relationship. Specifically, we calculated a kernel function to describe the relation between the eye tracking quantity and the EO-EPI envelope. We computed a kernel as follows. First, for each oculomotor time series we considered as meaningful oculomotor ‘events’ the top 10% of the peak values in the given series. Second, we calculated the mean EO-EPI signal in the interval [−10, 10] seconds around those peak events. For each participant, the triggered mean was normalized to that participant’s absolute maximum value, in this way producing the participant’s event-triggered average (ETA). To maintain independence between estimation and testing, the kernels linking an eye-tracking measure to the EO-EPI signal were calculated using a leave-one-participant-out procedure. That is, for each participant the kernel was derived as the mean of the ETAs calculated from all other participants. This kernel was convolved with the (left-out) participant’s eye-tracking time series, and a correlation with EO-EPI computed. The resulting correlation values (32 in all) were then Fisher-Z transformed and analyzed on the group level using a *t* test.

We used a different approach to evaluate the relation between blinks and EO-EPI dynamics. The blink time series was sparse and binary, with ‘1’ coding blink presence. We downsampled this time series to consecutive 2.5-sec windows, assigning to each window the value 1 if at least one blink was coded in the original series. For each participant we computed a blink-related event-triggered average by averaging the EO-EPI data around each blink (as described above). To determine the statistical significance of blinks and EO-EPI, we evaluated the reliability of the ETAs across participants: we calculated for each participant the correlation between his/or own ETA and the average of the ETAs of all the other subjects. We then tested the distribution of these (Fisher-Z transformed) correlation values at the group level using a *t* test.

### Statistical Inference for fMRI Analyses

#### Correlates of eye-tracking metrics

We examined whole-brain correlations between RS activity and several eye tracking measures: *GazeX*, *GazeX*^2^, *vel_GazeX*, *vel_GazeX*^2^, *Pupil_size*, and *blink function*. The BOLD data modeled were the “cleaned” time series from which only typical artifact sources were removed. We implemented two modeling approaches: in the first, we resampled each eye-tracking measure of interest to the sampling resolution of the MR acquisition (0.4 Hz) and convolved the result with canonical HRF via AFNI’s *waver* function to construct a regressor. In the second, we used a finite impulse response (FIR) function modeling approach where the BOLD impulse response was estimated using six tent functions (using AFNI’s *tent* basis function). This approach does not assume a fixed shape. From these estimates, we averaged the first three beta coefficients (corresponding to 0–7.5 sec post eye-tracker dynamics) and propagated the value to a group-level analysis. Family wise error correction was implemented using FSL’s TFCE implementation.

#### Correlates of EO-EPI regressors

Beta values associated with EYE_*conv*_ or EYE_*raw*_ were transformed to MNI space. To identify clusters where these beta values were significantly positive or significantly negative, we computed voxel-wise statistics using a Wilcoxon signed-rank test, and then implemented cluster-level control for family-wise error using permutations as described below. We used a nonparametric test because the relevant beta values data did not satisfy typical parametric assumptions.

We defined statistically significant clusters as ones where the statistical significance (uncorrected) at the single voxel level was below *p* = .01, and where the cluster size (volume) passed a value determined from the sampling distribution we derived using the following permutation procedure. In each of 10,000 permutations, we reversed the signs of 42 of the 83 datasets, and we implemented a Wilcoxon signed-rank test (Siegel & Castellan, 1956) to identify all clusters consisting of voxels where the statistical significance of the difference from chance (zero; 0) exceeded *p* < .01 and where all values were positive (we limited to positive values so that the resulting clusters could not combine both negative and positive values, as our main analysis also probed for clusters where all values were either positive or negative). We saved the largest cluster size from each permutation, and the resulting set of 10,000 values of largest cluster sizes defined the sampling distribution. The 95% percentile rank entry of the sampling distribution served as the critical value. This value was used to define statistically significant clusters in the experimental data. In addition, in those clusters defined as statistically significant, we computed the voxel-level effect size of the test (see Poldrack et al., 2008). We used the effect size (*r*) definition for the Wilcoxon test, quantified as *r* = *Z*/√(*N*), where *N* is the number of participants (data pairs). To determine whether the clusters identified by the EO-EPI/BOLD analyses were differentially driven by the young or older participant groups, for each of the statistically significant clusters we compared the mean beta value per cluster between the two groups. For each participant, we extracted the mean beta from the EO-EPI/BOLD regression, per cluster. We then evaluated whether these values differed for older and younger participants (Mann-Whitney between groups nonparametric test).

To evaluate whether significant EO-EPI correlates were found in areas dominated by artifacts, we calculated voxel-level temporal signal-to-noise ratio (tSNR) for each participant. To create a tSNR map for each participant, we used the raw functional images (before applying any signal processing steps), but after removal of the initial 10 stabilization images. We divided the absolute mean value of each voxel by its standard deviation. We then applied the statistically significant clusters found for EYE_*raw*_ and EYE_*conv*_ series as masks to determine mean ± standard deviation of the tSNR in each statistically significant spatial cluster. The motivation for this analysis was a prior report (W. Chen & Zhu, 1997) showing that Nyquist ghosting artifacts can propagate eye signals into midbrain areas (in the case of axial acquisition). Two MR physicists examined the QA reports produced by the scanner and did not find evidence for ghosting. However, we still wanted to evaluate if any EO-EPI whole-brain correlates were found in regions with low tSNR.

To evaluate the specificity of our findings to the eye-orbit region, we defined a control region of interest (ROI) in the maxillary sinus cavity below the eye, and analyzed the mean time series of that region identically to how we analyzed the data from the eye orbit region. Given the axial acquisition, ghosting is not likely to be propagated to this more inferior region.

In addition, we evaluated the relation between the EO-EPI regressor and the framewise-displacement regressor to understand the contribution of the latter to the EO-EPI data. We computed the correlation between the FD regressor and EYE_*raw*_ regressor per person, normalized the correlation values (Fisher-Z) and conducted a statistical test at the group level. We conducted a similar analysis to evaluate the relationship between EO-EPI and the global signal (GS). We defined GS as the mean whole-brain time series of all gray matter voxels, following removal of the motion artifacts, WM/CSF contributions, and subsequent to spatial smoothing. Because GS also contains neural information (e.g., Liu, Nalci, & Falahpour, 2017) we did not partial out GS, but evaluated its relationship to EO-EPI. We used the same approach we applied to framewise displacement.

To study the relation between EO-EPI activity and regions previously linked to oculomotor control, we defined the frontal eye fields (FEF) and supplementary eye fields (SEF) as independent ROIs and for each we examined correlations with the EO-EPI regressor. To create FEF and SEF ROIs, we used the NeuroSynth database (Yarkoni, Poldrack, Nichols, Van Essen, & Wager, 2011). The probability mask corresponding to the keyword *eye* was saved and thresholded by z-score of 7 (max Z = 9.1, generated from 417 studies). From the thresholded image, regions around the intersection of precentral sulcus and superior frontal sulcus were marked as FEF, and a region around the medial frontal gyrus was marked as SEF (see Supporting Information Figure S6). Those masks were spatially translated to the individual-subject space and mean activation of those two ROIs extracted from the cleaned and smoothed data. We constructed a regression model to predict the FEF and SEF ROIS’ activity from the EO-EPI series, per participant. Coefficients were analyzed using a Wilcoxon rank sum test.

#### Functional connectivity maps and derived network metrics

To create functional connectivity networks, we used a resting-state functional connectivity parcellation based on 500 ROIs (Schaefer et al., 2018). We spatially translated this parcellation into each participant’s individual space, where they were further limited to gray matter by multiplying all ROIs with the participant-specific gray matter mask (to limit the influence of data from nongray matter areas). We extracted the mean time series from each ROI, for the two types of spatially smoothed resting-state data we derived (one typical, and the other with EO-EPI EYE_*conv*_ regressed). We examined the network features after thresholding the connectivity matrices at 12 sparsity levels: 30%, 20%, and 1–10%. In all, from each participant’s resting-state network we derived the following metrics: node degree, strength, cluster coefficient, transitivity, assortativity, efficiency, number of communities, betweenness centrality, and modularity. Subsequent to thresholding, the feature values were processed as follows. We generally used nonbinarized connections maintaining the original weights, with the following exceptions: (a) for node degree we used binarized values; (b) for clustering coefficient, transitivity and betweenness centrality we used normalized values, per participant, per condition; (c) for betweenness centrality we used connection-length matrices as inputs. We calculated these using the Brain Connectivity Toolbox (Rubinov & Sporns, 2010) (see Supporting Information for description of the metrics as described in the Brain Connectivity Toolbox). We calculated these parameters for the original and “clean” networks as defined above. We then tested which of these parameters differed as a result of the EO-EPI removal procedure using paired-sample *t* tests. We defined a robust result as one that was statistically significant across all 12 levels of sparsity thresholding. We report the results for all network metrics for completeness so that they could be cross-referenced again prior and future work. Because subsets of those features are expected to be correlated, we constructed correlation matrices (using across-participant variance) to identify positive and negative correlations between features in order to inform our discussion of changes to modularity.

We also probed for changes in global topology by quantifying the impact of EO-EPI removal on the shape of the entire degree distribution (for the largest three sparsity levels: 10%, 20%, 30%). Following prior work (e.g., Fornito, Zalesky, & Bullmore, 2010) we fit an exponentially truncated power law function to each participant’s degree distribution. The function was *Y* = *a* × *X*^*b*^ × *e*^(*x*×*c*)^, where *Y* is the cumulative probability of the distribution and *X* is node degree. From this equation, we derived the coefficient (*a*), power law exponent (*b*), and degree cutoff point (*c*). A paired-sample *t* test was applied to each parameter to evaluate the impact of partialling out EO-EPI.

We wanted to know whether fronto-parietal systems that mediate exogenous attention become less hub-like when EO-EPI is removed. To evaluate this, we used previously defined criteria (Xu et al., 2014) in order to detect network hubs, separately for each of the three largest sparsity thresholds. These criteria required that the value of a node be higher than 1 *SD* above the mean value for each of these empirical distributions: node strength, node degree, and node betweenness centrality. Nodes matching all three criteria were considered hubs. The chance probability of a node being a hub (assuming a normal distribution) is ∼0.34^3^ = .04. To evaluate whether removal of EO-EPI variance impacted whether a region satisfied hub criteria, for each region we counted the number of participants for which the region was classified as a hub, with our without EO-EPI removal. On a binomial, a difference would need to consist of at least seven or more participants (binomial test parameters: *N* = 83; *K* = 7; *p* = .04).

We also identified any specific pair-wise differences in regional connectivity for the raw and cleaned matrices. After applying Fisher’s Z transformation, pair-wise correlation values were subjected to paired-sample *t* tests. We used false discovery rate (FDR) to correct for multiple comparisons.

#### Dual regression

We used dual regression to determine how removal of activity associated with the EO-EPI regressor impacted connectivity in previously defined resting-state networks. The procedure was implemented in AFNI and followed workflows described previously (Beckmann, Mackay, Filippini, & Smith, 2009; Nickerson, Smith, Öngür, & Beckmann, 2017). In the first step we used 14 predefined resting-state network spatial masks (Shirer, Ryali, Rykhlevskaia, Menon, & Greicius, 2012) to extract ‘seed’ time series for each of the networks. The 14 resting-state network masks were spatially transposed to individual space and multiplied by the gray matter mask of each participant to reduce contribution from nongray matter areas. For each participant we then produced two seed time series for each of the 14 networks: one from the functional data from which the EO-EPI variance was not removed, and one from the functional data from which this variance was removed using the EYE_*conv*_ regressor.

To determine whole-brain connectivity of the seed regions, we inserted all 14 time series into a single multiple regression. In effect, we conducted two separate regression models: Model #1 was a “typical” model where the mask-derived seed time series produced from the original (typically processed) functional data served as regressors to predict whole-brain resting-state data. This process reproduces the typical dual regression procedure. Model #2 was an “EO-EPI-removed” model. Here, the dataset analyzed was the EO-EPI-removed BOLD data. From that point on, the dual regression was carried out as usual, with seed time series (one per network) used conjointly to predict whole-brain activity.

The produced beta weights were analyzed using group level repeated-measures test to identify seed time series whose connectivity differed between the two data sets; that is, whose connectivity was impacted by the EO-EPI removal procedure. We used FSL’s *randomise* function (Jenkinson et al., 2012). A within group *t* test with 10,000 permutations and threshold-free cluster enhancement was applied. Because our interest was in evaluating the impact EO-EPI regressor we adopted a liberal approach of not correcting for multiple comparisons across the 14 networks tested in the dual regression procedure. We also note that the 14 time series used for dual regression were relatively weakly correlated in this data set: to determine collinearity, on the single participant level we computed the 14 × 14 cross-correlation matrix and then averaged these across participants. The highest mean correlation was 0.55, which licensed separate analyses for each network regressor.

#### Relation between analyses and control for multiple comparisons

Taken together, we report two core independent analyses: (a) the first uses the EO-EPI regressor for whole-brain analyses, using a convolved or nonconvolved regressor. Its findings constrain the findings from the pairwise functional connectivity analysis based on the 500-region parcellation, because regions identified by EO-EPI/BOLD are more likely to show reduced connectivity after removing the EO-EPI contribution; (b) the second analysis is the network-metric analyses: some of its findings (e.g., modularity, clustering) are independent of other analyses. The whole-brain analysis is corrected for family-wise error, whereas the network metric is not corrected for multiple tests in order to allow cross-referencing our network-level findings against prior and future literature. In addition, we report several analyses in order to offer insight into mechanisms, or for compatibility with prior studies. Specifically, the analyses of the relation between EO-EPI and eye-tracking data are meant to elucidate the sources of the EO-EPI signal, rather than to provide further information on the relationship between eye movement and brain activity. This analysis is internally corrected for family-wise error. The analysis relating eye tracking to BOLD/fMRI is presented as a contrast to the EO-EPI and for consistency with prior work.

## RESULTS

### Eye-tracking Data: Quality and Correlation with Whole-brain BOLD

**Figure 1.**
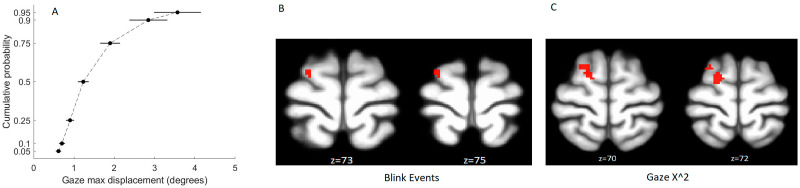
Relation between eye-tracking measures and EO-EPI regressor from eye orbits. (A) modest eye movements in 2-sec nonoverlapping time windows. (B) and (C) whole brain correlates of resting-state BOLD with blink events and *GazeX*^2^. No other areas showed statistically significant effects. Each analysis is corrected for multiple comparisons using FSL’s implementation of TFCE Family-wise error control.

Based on our artifact rejection criteria, usable eye-tracking data were available for 32 of 77 participants for which eye tracking data were collected. A power-spectra analysis of the eye tracking data (Supporting Information Figure S3) indicated higher broadband power in all frequencies in the rejected data, including those approaching the Nyquist frequency of the eye-tracking data in the current study (*f* = 30 Hz). Participants largely avoided making large eye movements during the resting-state session. To quantify these movements, we calculated the maximal displacement of gaze position in nonoverlapping 2-sec windows. The resulting empirical cumulative distribution functions (see Figure 1A) indicated modest movement, with around 50% of analysis windows showing displacement values below 1° and only around 10% of windows showing displacement values above 3°.

Whole-brain correlations with eye-tracking metrics were found for the *blink function* and *GazeX*^2^ regressors and presented in Figure 1B, C (*p* < .05, corrected for multiple comparisons with family-wise error; see Supporting Information Table S1 for coordinates). We note these findings were identified via a finite impulse response (FIR) analysis (see Methods) which estimated the HRF shape per regressor. Regressions based on canonical HRF-convolved regressors produced results that were not statistically significant.

### Eye-tracking Data: Correlation with Eye Orbit EPI Data

**Figure 2.**
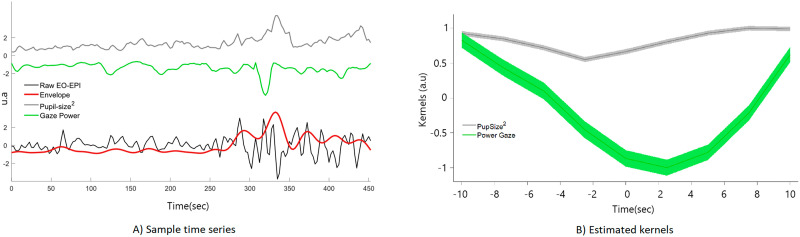
Relation between eye-tracking measures and EPI Orbit (EO-EPI) regressor. (A) sample time window showing relationship between raw EO-EPI signal, EO-EPI Envelope used for Kernel computation, and pupil size and gaze power measures derived from simultaneously acquired eye tracking data. (B) two Kernels estimated as relating the relationship between the EO-EPI envelope and pupil size (gray) or gaze power (green). Note that peaks in eye-tracking gaze power (time = 0) precede a peak in EO-EPI envelope by around 2 sec.

We evaluated the correlation between each of the 12 types of eye tracking time series (see Methods) and the EO-EPI data. We controlled for the 12 tests using Bonferroni correction, because some of the eye-tracking measures were highly correlated (see Supporting Information Figure S4). We identified three eye-tracking regressors that significantly predicted the EO-EPI envelope (Bonferroni corrected for 12 tests): the gaze power (*vel_GazeX*^2^ + *vel_GazeY*^2^), square of pupil size *PupilSize*^2^, and the gaze velocity in the vertical (*Y*) direction. The pupil size was evaluated as deviation from the subject’s mean value, so its squared value indicated absolute deviations from mean value. We used squared deviation rather than absolute value as the derivative of the exponent is better behaved than that of the absolute function. Figure 2A shows sample time series reflecting raw EO-EPI, its envelope, and eye-tracking regressors, and Figure 2B shows the estimated Kernels for gaze power and square of pupil size.

Pupil size squared explained 7 ± 2% of the variance of the EO-EPI envelope and presented a significant positive correlation: *ρ* = 0.17 ± 0.05, *t*(30) = 3.45, *p* = .0017, *d* = 0.62. Gaze power explained 5.4 ± 1.6% of the variance of the EO-EPI envelope and had a significant negative correlation: *ρ* = −0.17 ± 0.03, *t*(30) = 5.18, *p* < .001, *d* = 0.93. These two variables jointly explained the 11 ± 3% of EO-EPI envelope’s variance, a significant improvement in model performance with respect to the single variable cases (Δ*BIC* < −2). Gaze velocity in the *Y* direction had a weaker impact; it explained 3.7 ± 1.0% of the EO-EPI’s envelope variance and had a significant positive correlation: *ρ* = 0.11 ± 0.03, *t*(30) = 3.67, *p* < .001, *d* = 0.66. Adding this variable to the preceding regression model did not significantly increase explained variance (Δ*BIC* = −0.5). The exact numeric values corresponding to these kernels is given in Supporting Information Table S2. Blinks were not significantly correlated with EO-EPI.

### Connectivity of EO-EPI regressors

**Figure 3.**
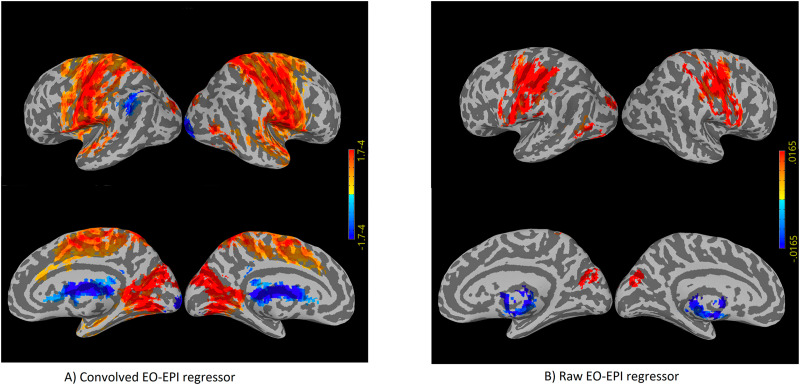
Whole-brain connectivity maps for the EYE_*conv*_ (A) and EYE_*raw*_ regressors (B). These were produced by deriving a mean time series from each participant’s eye orbit, correlating it with each voxel’s time series, and then producing family-wise error-corrected group-level maps using a single-voxel threshold of *p* < .01, and cluster correction based on permutations. ’Convolved’ refers to an analyses where the orbital time series was convolved with an HRF basis function, whereas ’Raw’ refers to nonconvolved regressors.

**Figure 4.**
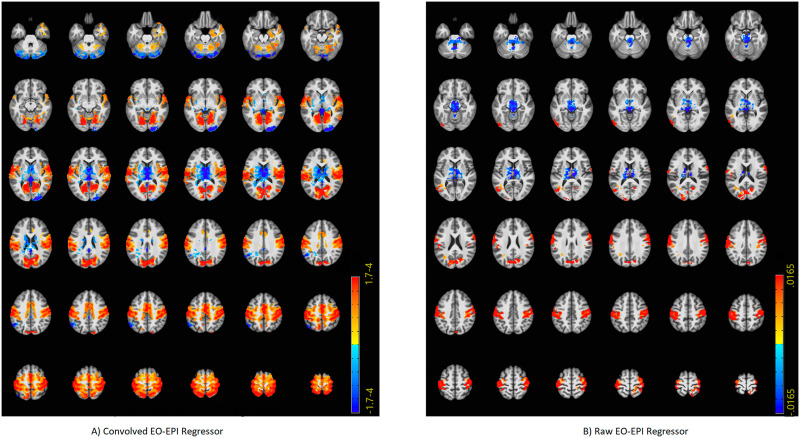
Axial slices showing whole-brain connectivity for the EYE_*conv*_ (A) and EYE_*raw*_ regressors (B). The figure reports the results of the same analysis depicted in Figure 3, but overlaid on axial slices.

We identified an extensive system that correlated with the EO-EPI regressor. For the convolved version of the EO-EPI regressor (EYE_*conv*_), we found correlations in pre- and postcentral gyri bilaterally, parts of the superior temporal gyrus and visual cortex (Figure 3A). We also identified strong correlations (of opposite sign) in the thalamus (Figure 4A). In addition, we identified whole-brain correlations for the nonconvolved versions of the EO-EPI regressor (EYE_*raw*_). These were qualitatively similar, but reduced in extent (see Figures 3B and 4B). Whole-brain clusters in MNI space for the EYE_*raw*_ and EYE_*conv*_ regressors are reported in Supporting Information Tables S3 and S4. We examined the effect size of the test for each voxel within these statistically significant clusters. As shown in Supporting Information Figure S7, effect-size values peaked at around 0.5 in sensorimotor and visual cortices. In addition, for each statistically significant cluster we evaluated whether correlations differed for younger and older participants, but no cluster showed a statistically significant result. A region of interest analysis indicated statistically significant correlations with EO-EPI in FEF (Wilcoxon *z* = 6.15, *p* < .001) but not in SEF (*z* = −1.28, *p* > .05).

An identical analysis that used time series from the maxillary sinus cavity rather than the eye orbit area produced a different pattern of results: the distribution of clusters was mainly limited to the sinus and eye areas with some ghosting presented along the *Z*-direction, as expected. The distribution does not resemble that found for the (nearby) eye orbit area (see Supporting Information Figure S9).

In general, the tSNR of the raw time series was quite good across the cortex (see Supporting Information Figure S8), with typical drop-off in low-signal areas and those susceptible to motion. Values were similar to the those reported by the Human Connectome Project for 2-mm and 3-mm noncleaned data (Smith et al., 2013). We treated each cluster where BOLD activity correlated with EO-EPI (raw or convolved) as a functional ROI and calculated the mean and standard deviation of tSNR in each cluster across participants. Most of these areas were associated with adequate tSNR, including the thalamus. This held for all statistically significant clusters picked up by the EYE_*raw*_ regressor (see Supporting Information Table S5). For EYE_*conv*_ the clusters found in the left and right cerebellum were associated with low tSNR (and relatively systematically across participants, see Supporting Information Table S6), as was a cluster in the mid occipital gyrus bilaterally (potentially as it includes time series from the field of view between the two hemisphere).

### EO-EPI Regressor: Variance, Power Spectra, and Relation to Motion Parameters and Global Signal

**Figure 5.**
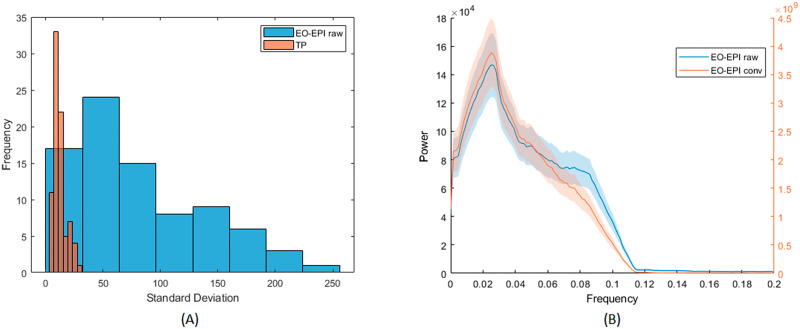
Spectral and spread properties of EO-EPI regressor. (A) Across-participant distribution of standard deviations of EO-EPI time series and (for comparison) average time series from temporoparietal junction ROI. (B) Frequency distribution of convolved and raw EO-EPI series. Differences in order of magnitude are due to convolution with HRF basis function.

Across participants, the time series of the EO-EPI regressor presented a larger range of standard deviation values than found in other ROIs. Figure 5A presents a histogram of the standard deviation values for EYE_*raw*_ in the participant group and comparative values from the temporoparietal junction (TPJ). The standard deviation values for TP were relatively low and tightly clustered in the range of 5–45, with a mode of 10. In contrast, for the EO-EPI regressor, there was much less systematicity in the spread of values across participants: the distribution of standard deviation values was relatively more uniform and showed much larger values, some with *SD* > 200. The mean number of voxels in these regions was 1,270 for TPJ and 406 for EYE_*raw*_.

The reason for these differences across participants is unclear. However, a byproduct is that when the EO-EPI regressor is correlated with brain activity in the context of regression, the resulting beta values for this regressor have a very broad distribution with significant differences across participants and outliers. For this reason, using a parametric test on the group level can produce false negatives or positives. To illustrate, in this current study, when nonparametric tests are used for group-level analysis, then both the Sign test and the Wilcoxon test produce group-level significance maps as reported here. AFNI’s multilevel analysis *3DMEMA* (G. Chen, Saad, Nath, Beauchamp, & Cox, 2012), which downweights beta values from participants with noisier beta estimates, produces similar results, though statistically weaker. However, a typical group-level *t* test of beta values against zero produced a null result.

The large standard deviation of the EO-EPI regressor was related to peaks in that signal. As indicated in the Methods section, applying a ‘despiking’ procedure reduced the sensitivity of the whole-brain correlation analysis: its most extreme effect was flattening several time series from the eye orbit area, and in other cases it impacted a large number of time points in that area (see Supporting Information Figure S5 for illustration). An analyses of the spectral features of EO-EPI (Figure 5B) showed a strong peak in those time series at 0.04 Hz, that is, a cycle of 25 sec. This is consistent with slow fluctuations sometimes observed in cortical regions. To summarize, the EO-EPI regressor, as would be expected, presented some time domain features (spikes and strong interindividual differences in spread) that differ from BOLD time series acquired in the brain, and these need to be considered during preprocessing and group-level analyses. That said, its spectral power presented a strong peak at low frequencies of the sort seen for cortical BOLD time series.

With rare exceptions, EYE_*raw*_ was not correlated with the estimated head motion parameters. Significant correlations with any of the six motion parameters were found for 3 of the 83 participants: in the first case there was correlation with L/R displacement; in the second case there was correlation with L/R displacement and rotation; in the third case five of the six parameters were correlated. In all cases, correlation values were below 0.2. This lack of correlation suggests that variance in EYE_*raw*_ signal is not related to head motion, though an extreme case of movement may be picked up in this signal as well. We also examined if the EYE_*raw*_ EO-EPI regressor reflected framewise displacement, as well as its relation to the global signal (defined as mean gray matter signal after removal of motion, WM, and CSF regressors; see Methods). For framewise displacement the group-level test on Fisher-Z normalized correlation values indicated mean (and mode) value very close to zero (*M* = 0.006, *SD* = 0.14) producing a result that was not statistically significant at the group level, *t*(82) = 1.75, *p* > .05. For global signal the mean (Z-normalized) correlation was statistically significant at the group level, *t*(82) = 2.61, *p* < .01, but the absolute mean Fisher-Z value was still close to zero (*M* = 0.04, *SD* = 0.14), which corresponds to a mean Pearson’s *R* value of around 0.04.

### Functional Connectivity Networks

An analysis of the network metrics revealed that several were significantly impacted by EO-EPI removal, across all sparsity thresholds. The raw connectivity matrices presented higher values for node strength (both maximum and mean), and mean cluster coefficient (and transitivity). Conversely, maximized modularity was greater for the clean (EO-EPI-removed) matrices. Difference values, effect sizes, and results of statistical tests are reported in Table 1 and in Supporting Information File 1. As shown in the Tables, statistically significant results were associated with medium effect sizes in the range of 0.4–0.5. These results maintained almost without exception for networks at sparsity levels of 0.01 to 0.09 (see Supporting Information File 1). Supporting Information Table S7 reports the raw values for each metric, for the sparsity levels of 10%, 20%, 30%. In addition, we determined if age modulated the impact of EO-EPI removal on network metrics. We computed for each person the impact of EO-EPI removal for each network property and then tested if these values differ between age groups. None of the tests were significant. An across-participant correlation analysis indicated that modularity was generally negatively correlated with measures that load on stronger connectivity, including degree, strength and clustering coefficient (see Discussion).

**Table 1.** Difference of network metrics between Raw and Clean (EO-EPI-removed) functional connectivity matrices.

	**Sparsity = 0.1**			**Sparsity = 0.2**			**Sparsity = 0.3**		
	**Preserved nodes = 1,248**			**Preserved nodes = 2,495**			**Preserved nodes = 3,743**		
	Difference	Cohen’s D	T-stat	Difference	Cohen’s D	T-stat	Difference	Cohen’s D	T-stat
Max Degree	1.23	0.38	3.40**	0.74	0.37	3.33**	−0.04	0.02	−0.22
Min Degree	3.45	0.04	0.38	−2.58	0.08	−0.76	−1.90	0.10	−0.92
Max Strength	2.45	0.49	4.41***	2.11	0.46	4.13***	1.91	0.42	3.79***
Min Strength	8.03	0.10	0.86	−4.59	0.16	−1.47	−2.51	0.14	−1.26
Mean Strength	0.99	0.49	4.36***	1.22	0.46	4.14***	1.38	0.44	3.94***
Max Cluster Coefficient	0.50	0.12	1.06	1.21	0.26	2.32*	1.87	0.32	2.90**
Min Cluster Coefficient	6.05	0.02	0.14	−3.52	0.08	−0.70	−1.43	0.07	−0.61
Mean Cluster Coefficient	1.08	0.46	4.11***	1.65	0.47	4.22***	1.84	0.44	3.94***
Transitivity	1.92	0.46	4.11***	2.39	0.46	4.14***	2.49	0.45	3.99***
Assortativity	0.31	0.06	0.54	1.44	0.22	1.95	2.12	0.28	2.46*
Efficiency	0.12	0.18	1.60	0.58	0.49	4.39***	0.80	0.46	4.07***
Max Number of Community	0.02	0.01	0.10	−0.02	0.05	−0.42	−0.02	0.05	−0.44
Maximized modularity	−0.007	0.44	−3.95***	−0.005	0.38	−3.36**	−0.003	0.34	−3.03**
Max betweenness centrality	−0.21	0.02	−0.16	0.12	0.01	0.10	0.65	0.07	0.63
Mean betweenness centrality	0.86	0.41	3.70***	0.76	0.39	3.50***	0.25	0.14	1.29

*Note*. Differences shown are in units of percentage apart from the number of communities and maximized modularity which maintain the original scale; *p < .05, **p < .005, ***p < .001.

**Figure 6.**
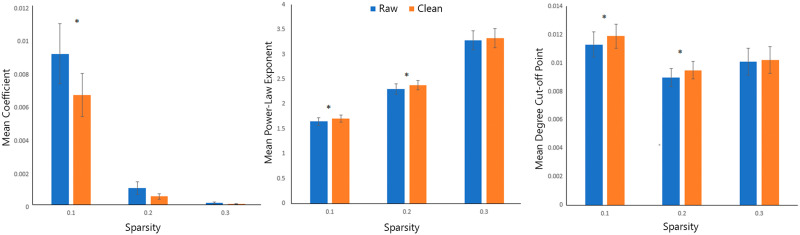
Analysis of degree distributions. Degree distributions were fit using an exponentially-truncated power law with three parameters: coefficient, power law exponent, power law cutoff point. The three bar plots show the mean values of these parameters across the three largest sparsity levels. Bar pairs for which a difference was significant are marked with a star (*). For sparsity of 0.1, all three parameters differed between raw and clean (EO-EPI removed) connectivity matrices.

Fitting the degree distributions using an exponentially truncated power law showed that the EO-EPI removed networks differed in the degree distribution (see Figure 6). As shown in Figure 6, for 10% sparsity networks, EO-EPI removal impacted all three coefficients of the truncated power-law fit: power law coefficient: *t*(82) = 3.33, *p* < .01, *d* = 0.37, power law exponent, *t*(82) = −3.70, *p* < .001, *d* = 0.41, and degree cutoff point, *t*(82) = 3.59, *p* < .001, *d* = 0.4. For the 20% sparsity networks, differences were found for power law exponent, *t*(82) = −3.13, *p* < .01, *d* = 0.37, and degree cutoff point, *t*(82) = 2.59, *p* < .01, *d* = 0.33. No statistically significant differences were found for 30% sparsity networks. Supporting Information Figure S10 presents mean degree distributions for Raw and Clean networks for these sparsity levels.

**Figure 7.**
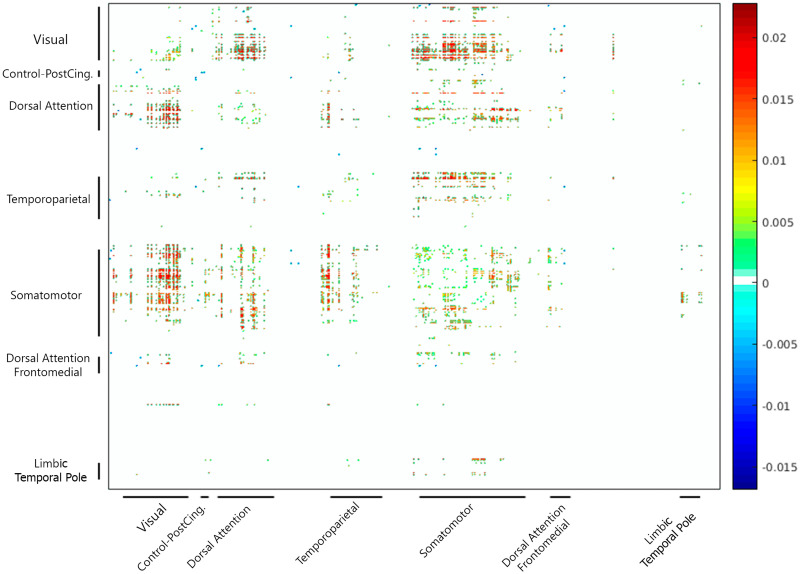
Impact of EO-EPI removal on pairwise connectivity. For each participant, 500-region connectivity matrices were produced from time series from which the variance attributable to EO-EPI was either removed (‘clean’) or not (‘raw’). Pairwise connectivity differences were then computed at group level to identify region pairs where EO-EPI removal produced a change in connectivity strength. Family-wise control: *Raw* − *Clean*, *p* < .05 two tailed, for each single connection, corrected for multiple comparisons using FDR.

We determined which areas tended to show changes in connectivity as a function of EO-EPI removal. In general, this analysis is not independent of the whole-brain correlation with the EO-EPI time series used as a regressor, but it is more sensitive in identifying strongest pairwise differences. For each of the 124,500 pairwise correlations we conducted a *t* test to determine whether the pairwise correlations differed for raw and EO-EPI-removed connectivity matrices. The results (FDR corrected; Figure 7) showed that connectivity matrices constructed from the raw matrices presented stronger connectivity between sensorimotor areas and temporoparietal, dorsal attention, visual cortex, and other sensorimotor regions. There were relatively few regions that showed stronger connectivity in the EO-EPI-removed condition, notably the posterior cingulate, which showed stronger connectivity with multiple other brain areas.

The dual regression analysis did not identify any predefined RS network for which connectivity changed significantly. A hub-focused analysis that examined whether there were regions more frequently identified as hubs in the raw or EO-EPI-removed series also produced a null result: the most extreme example was a region defined as hub for 20 participants in one case and 25 in another (a nonsignificant difference on a binomial). While the location of these hubs was not a central point of the current study, broadly speaking, for the 10% sparsity threshold (raw) matrices, hubs were localized to motor and sensorimotor areas (9 regions) Dorsal attention (6 regions), DMN (4 regions), temporal-parietal areas (4 regions), and ventral attention (2 areas). Only one visual extrastriate area was identified as a hub.

## DISCUSSION

Neuroimaging is continuously expanding our understanding of the principles that determine organized patterns of RS connectivity. Our findings demonstrate that endogenous eye movements during RS contribute significantly to structured patterns of RS connectivity. Our main finding is that eye movements, measured via EPI time series recorded from the eye orbits, identified a sensorimotor system that appeared to be linked to oculomotor activity. Removal of activity accounted for by eye movements had systematic impact on whole-brain connectivity. We first address issues related to oculomotor measurement during the resting state that emerged in the study and then discuss the implications of the results for basic and applied research.

### Probing Resting-state Networks with Eye Tracking and Eye Orbit EPI Data

As reviewed in the Introduction, few studies have studied brain activity patterns that are correlated with oculomotor activity during the resting state, and those have produced inconsistent and sometimes puzzling results. The most relevant is Fransson et al. (2014, *N* = 18): It derived gaze velocity data from eye tracking during a resting-state scan, finding correlation with DMN activity. Also related is McAvoy et al. (2012, *N* = 9), which examined Brain/EOG correlations and reported a null result. In our own analyses of eye tracking data (*N* = 32), we found correlation between BOLD-RS and only two eye tracking metrics: horizontal eye displacement and blinks. These relatively modest correlations could be the result of noise in the eye tracking data, which presented itself in higher power across all frequencies for rejected data as compared to analyzed data. We also note that participant exclusion for the eye tracking data was more extensive in the older age group, and so future studies of related topics could prefer to collect data from younger participants unless there is a specific interest in the older population.

We found correlations between the eye tracking metrics and EPI data recorded from the eye orbit area (EO-EPI), Bonferroni corrected for 12 correlation tests. These were found for gaze power, pupil size (squared), and gaze velocity in the *Y* (horizontal) direction. These data are consistent with several prior reports. Beauchamp (2003) showed that peaks in the EO-EPI time series occur when an MR acquisition coincides with a rapid saccadic eye movement. Brodoehl, Witte, and Klingner (2016) and Son et al. (2019) showed that EO-EPI data can be used to estimate gaze location (when nonaveraged; i.e., used in a multivariate context). In addition, Beauchamp’s observations suggest that for our interleaved acquisition, eye movements occurring either during odd- (up direction) or even-numbered (down direction) slice acquisition could be picked up in the analysis, because we treated the entire eye orbit as a single ROI. Consequently, while the volume acquisition time was 2.5 sec, our effective temporal resolution for the eye orbit ROI could have been higher, as we could identify eye movement during both the up- or down-acquisition direction. EO-EPI fluctuations are likely mainly driven by signal disturbances due to air/tissue motion, but we cannot exclude the possibility that the signal also contains a BOLD component, due to the metabolic activity in nearby muscles. In particular, Law (1998) used PET rCBF to study brain systems involved in generation of voluntary saccades and reported active areas in the eye orbit, “primarily located close to the apex of the pyramidal shaped orbital cavity.” Our finding of a systematic delayed coupling in which changes in gaze power preceded local minima in EO-EPI fluctuations (the latter delayed by ∼2 sec), and of a strong peak frequency of 0.04 Hz for EO-EPI are both consistent with the possibility that EO-EPI also reflects metabolic activity. We also found little independent evidence to suggest a strong contribution of motion artifacts to EO-EPI: beyond one participant for which five of six motion parameters correlated with EO-EPI, we only found two additional correlations with motion elements, for two additional participants. In addition, regarding framewise displacement (FD), this regressor too was removed prior to the EO-EPI analysis, and separately, we found no systematic relation between FD and EO-EPI on the single-participant level. With respect to relation to Global Signal (derived here from gray matter), we found a statistically significant relation with EO-EPI, but the absolute magnitude of correlation was modest with mean Pearsons’s *R* value of around 0.04. A modest component of GS could therefore be related to eye movements.

Note that task compliance during this RS study was good. First, participants were continuously monitored and experimenters verified participants did not drift off to sleep during the scan. Second, the eye tracking data indicated compliance with the task instructions in that the eye movements that were made during fixation were modest in magnitude (see Figure 1A). When evaluating average eye movement between successive 2 sec epochs, we found that in 75% of the cases, the magnitude was below 2°, which corresponds to a small displacement. For this reason, we consider these data to be representative of typical compliant behavior during wakeful rest.

Given these findings, it can still be asked whether, practically, one should control for oculomotor influences measured by EO-EPI in future work. On the basis of these findings we suggest that EO-EPI should not be treated as a nuisance factor with the exception of very specific circumstances. In contrast to factors such as head motion that are a nuisance factor that complicate studying BOLD functions related to neural activity, EO-EPI/BOLD correlates do not appear to be spurious or necessarily linked to nonneural causes. For this reason, EO-EPI covariance should be maintained in the data, unless one has a very specific interest in those facets of brain connectivity (or dynamics) that are completely unrelated to the function of the brain’s motor systems. Otherwise, EO-EPI should be treated as an identifiable independent factor that is informative with respect to the natural function of oculomotor systems.

### Brain Systems Identified by Eye Orbit EPI (EO-EPI) Regressor

When used as a whole-brain regressor, the EO-EPI time series correlated with an extensive bilateral sensorimotor system. In addition, activity was found in superior parietal lobule, the dorsal part of the superior frontal gyrus, supplementary motor areas, and the extrastriate cortex in occipital lobe (excluding striate cortex). There was no indication for differences between younger and older participants in these areas. ROI analyses indicated activity in frontal eye fields. The topography of this system does not match either the ventral or dorsal attention networks as usually defined, but it is quite similar to the frontal-eye-field connectivity map reported by Fox, Corbetta, Snyder, Vincent, and Raichle (2006). It is also highly similar to activity maps reported for simple eye movements in absence of attention, which have identified extensive activity in motor and premotor areas (e.g., Balslev et al., 2011) with little fronto-parietal involvement. A subset of these regions was also picked up by a nonconvolved (‘Raw’) version of the EO-EPI regressor which may indicate that activity in these areas does not precede eye movements, but is relatively contemporaneous with them (to the extent that can be inferred from fMRI), or even that the eye movements reflected in the EO-EPI time series follow activity in those areas.

The brain areas we identify using EO-EPI (or eye tracking regressors) depart from ones frequently mentioned in studies of saccadic mechanisms, which prototypically reveal involvement of FEF/SEF and IPS. There are several possible explanations for this, which are not mutually exclusive. First, neuroimaging studies of saccades study saccade execution under exogenously determined conditions. Specifically, a distinction is made between two saccade categories, both externally controlled: ‘reflexive’ saccades that orient to peripheral (typically sudden) target appearance, and ‘voluntary’ saccades that are not oriented toward a target in an unmediated manner but rather require a cognitive judgment prior to eye movement (for review, see Mort et al., 2003). These voluntary saccades are studied by paradigms such as antisaccades (saccading to the opposite screen side of a target), memory-guided saccades (saccading to a location maintained in memory), or saccading to a location precued by an arrow. Note that both reflexive and voluntary saccades are associated with few degrees of freedom with respect to the actual saccade target, which constitutes a fundamental difference from the resting-state case. In addition, as indicated by Brown et al.’s study (reviewed in the Introduction), activity in FEF/SEF/IPS may not be related to oculomotor control per se, but to the paradigm demands that require attention and detection of visual cues. In support of this possibility, a recent study (Agtzidis, Meyhöfer, Dorr, & Lencer, 2020) examining eye movements during naturalistic movie viewing similarly failed to identify a frontal parietal system related to saccades (neither dorsal nor ventral attention systems; see their Table 2), but instead documented saccade-related activity in visual cortex, and smooth-pursuit activity in precuneus, cingulate, and occipital cortices. The authors attribute this failure to differences in paradigm, suggesting that natural viewing is associated with constant engagement rather than phasic shifts between fixation and saccades. This is also corroborated by a report by Son et al. (2019, *N* = 5) showing that during naturalistic viewing, data acquired from the eye orbits correlates with brain activity in areas that do not resemble the topography of attentional networks (see their Figure 5).

Another possibility, which does not assume substantial differences between RS and active tasks, is technical in nature. It is possible that endogenous oculomotor-linked sensory motor activity during resting state is simply not often reported just because fixation is frequently used as an implicit baseline in many oculomotor studies. If the network we identify is correlated with oculomotor activity both during fixation and saccade-to-target epochs (either reflexive or voluntary), then it will not be identifiable in analyses against baseline because it is partialled out in the contrast.

### The Impact of Removal of EO-EPI Properties from BOLD Activity

We examined the impact of removing the variance related to EO-EPI from brain activity using a few well-defined topographical and topological properties. For topography we found that removal did not have a statistically significant impact on connectivity in any of the 14 well-defined resting-state networks. We also examined the impact of removal on pair-wise regional connectivity using a 500-ROI parcellation (Schaefer et al., 2018). We grouped these 500 regions into 7 main clusters for purposes of graphical presentation (see Figure 7). The analysis produced statistically significant effects (FDR corrected), mainly showing that EO-EPI-removal was associated with reduced connectivity between the somatomotor regions and visual, temporoparietal, and also few dorsal-attention network areas. Also as shown in Figure 7, connectivity within each system was weakly impacted by EO-EPI removal if at all (i.e., few changes along the diagonal), which is consistent with the dual-regression results. To conclude, EO-EPI-removal appeared to primarily impact cross-network connectivity rather than within-network connectivity. Finally, we did not find evidence that EO-EPI removal impacted the distribution of network hubs in the brain.

However, robust results were found for both global and local topological metrics identified by a network analysis, and we found no evidence that these differed for the younger and older participants. Here we address findings that were consistent across the three largest sparsity thresholds: 10%, 20%, and 30% of connections. For global properties, we find that modularity (Q) was higher for the clean matrices. We note that, across participants, modularity negatively correlated with local properties including degree, strength. and clustering coefficient. It may be that the finding of reduced modularity for clean matrices owes to its relation to certain other connectivity measures. One specific possibility is that weaker connectivity necessarily produces lower modularity. This, however, seems not to be the case, as it has been shown that periods of high modularity can be found for epochs of both very high and very low connectivity (Betzel, Fukushima, He, Zuo, & Sporns, 2016).

For local properties, we found that the raw matrices were associated with greater node-strength values (indicating sum of connectivity linked to each node). For max strength, the difference was 2.45% (effect size = 0.49). The mean cluster coefficient (and strongly related, transitivity) were also impacted, showing reduced values (approaching 2.5% difference; effect size = 0.49) for the cleaned time series.

These changes are consistent with our other findings. EO-EPI is correlated with occipital, sensorimotor and few fronto-parietal areas, and as shown, EO-EPI removal predominantly impacts interregional/interinternetwork connections rather than intranetwork connections. For this reason, its removal serves to increase the modularity of resting-state networks.

### Implications for Network Studies of Typical and Apecial Populations

Graph theoretical approaches are increasingly applied in the context of resting-state fMRI studies of clinical disorders (Hallquist & Hillary, 2018). In some cases, these features are deployed clinically to define new clinical subtypes, and in other cases, they are used to advance understanding of the brain systems that may be associated with the clinical deficit. Being able to link differences in graph-theoretic metrics to the oculomotor systems can increase the specificity of the explanations provided by RS analyses, by linking differences to a specific behavior. It could also allow determining to what extent differences in RS connectivity between populations can be attributed to differences in oculomotor activity during resting-state acquisition.

A number of examples present the logic of this approach. For example, Parkinson’s disease (PD) is associated with changes to functional connectivity when analyzed both from dynamic and static perspectives (Kim et al., 2017). Neurophysiologically, it is associated with abnormality in eye movement control, including the generation of voluntary saccades. Anomalies are more evident for voluntary saccades, in early stages of disease (for review, see Pretegiani & Optican, 2017). A behavioral study (Zhang et al., 2018) showed that PD is linked to reduced fixation stability when fixation is required. Conversely, during free viewing of single images, PD patients make fewer saccadic eye movements, and within a more narrow range. Differences in network modularity for clinical populations have been documented in the case of autism, which present lower modularity (Rudie et al., 2013) and traumatic brain injury (Han et al., 2014), which has been associated with higher modularity and lower participation coefficient of sensorimotor systems (i.e., these areas are more weakly involved in between-module connectivity). In addition, schizophrenia (e.g., Alexander-Bloch et al., 2012) has been linked to changes in RS connectivity. Alexander-Bloch et al. showed that schizophrenia is associated with reduced modularity in functional networks, with motor areas bilaterally linked to different partitions. Individuals diagnosed with schizophrenia show lower mean saccade frequency during free gaze (Dowiasch et al., 2016) and during free viewing of photos, their gaze is limited to smaller areas of the photo (e.g., Morita et al., 2020; Silberg et al., 2019).

Our findings could also have implications for the study of dynamic, time-varying connectivity in healthy and clinical populations. Knowing that some dynamic changes are associated with phasic states of eye movements would allow better interpretation of the drivers of time-varying dynamics. An early study of time-varying dynamics (Hutchison, Womelsdorf, Gati, Everling, & Menon, 2013) is consistent with this possibility. It documented time points presenting phasic, strong connectivity between frontal eye fields, sensorimotor regions and occipital regions, whereas such connectivity was completely absent at other time points. This suggests temporary synchronization of multiple brain networks in relation to eye movement.

### Conclusions

We found that oculomotor movement provides a systematic contribution to RS connectivity in the human brain. It is correlated with activity in a brain network that largely involves sensorimotor and visual cortex, as well as the frontal eye fields. Removal of oculomotor contribution, as quantified via EPI time series sampled from the eye orbit area, produces changes to global topological features of RS networks. Isolating this contribution can produce a better understanding of activity sources that organize RS networks in health and disease, and could improve the use of RS network features in the context of machine learning.

## ACKNOWLEDGMENTS

The authors are grateful to the Sleepy Brain project team for openly sharing the data, to staff physicist Rouslan Sitnikov for technical advice, and to Jorge Jovicich for assistance in assessment of ghosting artifacts.

## SUPPORTING INFORMATION

Supporting information for this article is available at https://www.doi.org/10.1162/netn_a_00186.

## AUTHOR CONTRIBUTIONS

Cemal Koba: Formal analysis; Investigation; Methodology; Visualization; Writing – original draft; Writing – review & editing. Giuseppe Notaro: Formal analysis; Methodology; Visualization; Writing – original draft; Writing – review & editing. Sandra Tamm: Data curation; Investigation; Resources; Writing – review & editing. Gustav Nilsonne: Data curation; Investigation; Resources; Writing – review & editing. Uri Hasson: Conceptualization; Investigation; Methodology; Supervision; Writing – original draft; Writing – review & editing.

## FUNDING INFORMATION

Gustav Nilsonne, Riksbankens Jubileumsfond (https://dx.doi.org/10.13039/501100004472), Award ID: P15-0310:1. Gustav Nilsonne, Fredrik och Ingrid Thurings Stiftelse (https://dx.doi.org/10.13039/501100003186), Award ID: 2014-00037, 2015-00170.

## TECHNICAL TERMS

Electrooculography (EOG): A technique for measuring eye position using two electrodes that record the variation in electric potential generated by the eye displacement.
Pupil size: A measure of the dilation of a participant’s pupil. Usually expressed as the length of the major axis of the ellipse that best approximates the pupil, normalized to the camera’s sensor size.
Global network metrics: Network metrics that derive from the entire (global) network structure.
Local network metrics: Network metrics quantifying characteristics defined by relations between local units in the network.
Wilcoxon signed-rank test: A nonparametric statistical test that can be applied to repeated-measurement designs.
tSNR map: A parameter map showing tSNR values for each brain area, defined as the ratio of the mean of a signal divided by its standard deviation.
Exponentially truncated power law: A distribution that follows a power law multiplied by an exponential decay.
Dual regression: An ICA-based technique that allows creating participant-specific connectivity maps from network templates defined in standard space or group template.
Convolution: Mathematical transformation of a function through the weights provided by a second function that is referred to as a ‘kernel.’ This operation can be conducted both in time, space, or both.
Time-varying connectivity: An approach for studying time-varying aspects of functional connectivity.

## References

[bib1] Agtzidis, I., Meyhöfer, I., Dorr, M., & Lencer, R. (2020). Following Forrest Gump: Smooth pursuit related brain activation during free movie viewing. NeuroImage, 116491. https://doi.org/10.1016/j.neuroimage.2019.116491, 31923604 31923604 10.1016/j.neuroimage.2019.116491

[bib2] Alexander-Bloch, A., Lambiotte, R., Roberts, B., Giedd, J., Gogtay, N., & Bullmore, E. (2012). The discovery of population differences in network community structure: New methods and applications to brain functional networks in schizophrenia. NeuroImage, 59(4), 3889–3900. https://doi.org/10.1016/j.neuroimage.2011.11.035, 22119652 22119652 10.1016/j.neuroimage.2011.11.035 PMC3478383

[bib3] Avants, B. B., Tustison, N., & Song, G. (2011). Advanced Normalization Tools (ANTS) Brian B. Avants, Nick Tustison and Gang Song, 1–35. Retrieved from www.picsl.upenn.edu/ANTS.

[bib4] Balslev, D., Albert, N. B., & Miall, C. (2011). Eye muscle proprioception is represented bilaterally in the sensorimotor cortex. Human Brain Mapping, 32(4), 624–631. https://doi.org/10.1002/hbm.21050, 21391252 21391252 10.1002/hbm.21050 PMC4962903

[bib5] Beauchamp, M. S. (2003). Detection of eye movements from fMRI data. Magnetic Resonance in Medicine, 49(2), 376–380. https://doi.org/10.1002/mrm.10345, 12541259 12541259 10.1002/mrm.10345

[bib6] Becker, W., & Fuchs, A. (1969). Further properties of the human saccadic system: Eye movements and correction saccades with and without visual fixation points. Vision Research, 9(10), 1247–1258. https://doi.org/10.1016/0042-6989(69)90112-6, 5360604 5360604 10.1016/0042-6989(69)90112-6

[bib7] Beckmann, C., Mackay, C., Filippini, N., & Smith, S. (2009). Group comparison of resting-state fMRI data using multi-subject ICA and dual regression. NeuroImage. 10.1016/s1053-8119(09)71511-3

[bib8] Betzel, R. F., Fukushima, M., He, Y., Zuo, X.-N., & Sporns, O. (2016). Dynamic fluctuations coincide with periods of high and low modularity in resting-state functional brain networks. NeuroImage, 127, 287–297. https://doi.org/10.1016/j.neuroimage.2015.12.001, 26687667 26687667 10.1016/j.neuroimage.2015.12.001 PMC4755785

[bib9] Birn, R. M. (2012). The role of physiological noise in resting-state functional connectivity. NeuroImage, 62(2), 864–870. https://doi.org/10.1016/j.neuroimage.2012.01.016, 22245341 22245341 10.1016/j.neuroimage.2012.01.016 PMC13374118

[bib10] Brodoehl, S., Witte, O. W., & Klingner, C. M. (2016). Measuring eye states in functional MRI. BMC Neuroscience, 17(1). https://doi.org/10.1186/s12868-016-0282-7, 27411785 10.1186/s12868-016-0282-7 PMC4944461 27411785

[bib11] Brown, M. R., Goltz, H. C., Vilis, T., Ford, K. A., & Everling, S. (2006). Inhibition and generation of saccades: Rapid event-related fMRI of prosaccades, antisaccades, and no go trials. NeuroImage, 33(2), 644–659. https://doi.org/10.1016/j.neuroimage.2006.07.002, 16949303 16949303 10.1016/j.neuroimage.2006.07.002

[bib12] Chen, G., Saad, Z. S., Nath, A. R., Beauchamp, M. S., & Cox, R. W. (2012). fMRI group analysis combining effect estimates and their variances. NeuroImage, 60(1), 747–765. https://doi.org/10.1016/j.neuroimage.2011.12.060, 22245637 22245637 10.1016/j.neuroimage.2011.12.060 PMC3404516

[bib13] Chen, J., Lewis, L., Chang, C., Tian, Q., Fultz, N., Ohringer, N., … Polimeni, J. (2020). Resting-state “physiological networks.” NeuroImage, 116707. https://doi.org/10.1016/j.neuroimage.2020.116707, 32145437 32145437 10.1016/j.neuroimage.2020.116707 PMC7165049

[bib14] Chen, W., & Zhu, X.-H. (1997). Suppression of physiological eye movement artifacts in functional MRI using slab presaturation. Magnetic Resonance in Medicine, 38(4), 546–550. https://doi.org/10.1002/mrm.1910380407, 9324320 9324320 10.1002/mrm.1910380407

[bib15] Cox, R. W. (1996). AFNI: Software for analysis and visualization of functional magnetic resonance neuroimages. Computers and Biomedical Research, 29(3), 162–173. https://doi.org/10.1006/cbmr.1996.0014, 8812068 8812068 10.1006/cbmr.1996.0014

[bib16] de Vos, F., Koini, M., Schouten, T. M., Seiler, S., van der Grond, J., Lechner, A., … Rombouts, S. A. (2018). A comprehensive analysis of resting state fMRI measures to classify individual patients with Alzheimer’s disease. NeuroImage, 167, 62–72. https://doi.org/10.1016/j.neuroimage.2017.11.025, 29155080 29155080 10.1016/j.neuroimage.2017.11.025

[bib17] Dowiasch, S., Backasch, B., Einhäuser, W., Leube, D., Kircher, T., & Bremmer, F. (2016). Eye movements of patients with schizophrenia in a natural environment. European Archives of Psychiatry and Clinical Neuroscience, 266(1), 43–54. https://doi.org/10.1007/s00406-014-0567-8, 25472882 25472882 10.1007/s00406-014-0567-8 PMC4723634

[bib18] Dubois, J., Galdi, P., Paul, L. K., & Adolphs, R. (2018). A distributed brain network predicts general intelligence from resting-state human neuroimaging data. Philosophical Transactions of the Royal Society B: Biological Sciences, 373(1756), 20170284. https://doi.org/10.1098/rstb.2017.0284, 30104429 10.1098/rstb.2017.0284 PMC6107566 30104429

[bib19] Fonov, V., Evans, A., McKinstry, R., Almli, C., & Collins, D. (2009). Unbiased nonlinear average age-appropriate brain templates from birth to adulthood. NeuroImage. 10.1016/s1053-8119(09)70884-5

[bib20] Fornito, A., Zalesky, A., & Bullmore, E. T. (2010). Network scaling effects in graph analytic studies of human resting-state fMRI data. Frontiers in Systems Neuroscience, 4, 22. https://doi.org/10.3389/fnsys.2010.00022, 20592949 20592949 10.3389/fnsys.2010.00022 PMC2893703

[bib21] Fox, M. D., Corbetta, M., Snyder, A. Z., Vincent, J. L., & Raichle, M. E. (2006). Spontaneous neuronal activity distinguishes human dorsal and ventral attention systems. Proceedings of the National Academy of Sciences, 103(26), 10046–10051. https://doi.org/10.1073/pnas.0604187103, 16788060 10.1073/pnas.0604187103 PMC1480402 16788060

[bib22] Fransson, P., Flodin, P., Seimyr, G., & Pansell, T. (2014). Slow fluctuations in eye position and resting-state functional magnetic resonance imaging brain activity during visual fixation. European Journal of Neuroscience, 40(12), 3828–3835. https://doi.org/10.1111/ejn.12745, 25302817 10.1111/ejn.12745 PMC4309478 25302817

[bib23] Hallquist, M. N., & Hillary, F. G. (2018). Graph theory approaches to functional network organization in brain disorders: A critique for a brave new small-world. Network Neuroscience, 3(1), 1–26. https://doi.org/10.1162/netn_a_00054, 30793071 30793071 10.1162/netn_a_00054 PMC6326733

[bib24] Han, K., Mac Donald, C. L., Johnson, A. M., Barnes, Y., Wierzechowski, L., Zonies, D., … Brody, D. L. (2014). Disrupted modular organization of resting-state cortical functional connectivity in US military personnel following concussive ‘mild’ blast-related traumatic brain injury. NeuroImage, 84, 76–96. https://doi.org/10.1016/j.neuroimage.2013.08.017, 23968735 23968735 10.1016/j.neuroimage.2013.08.017 PMC3849319

[bib25] Hayes, T. R., & Petrov, A. A. (2016). Mapping and correcting the influence of gaze position on pupil size measurements. Behavior Research Methods, 48(2), 510–527. https://doi.org/10.3758/s13428-015-0588-x, 25953668 25953668 10.3758/s13428-015-0588-x PMC4637269

[bib26] Honey, C., Sporns, O., Cammoun, L., Gigandet, X., Thiran, J.-P., Meuli, R., & Hagmann, P. (2009). Predicting human resting-state functional connectivity from structural connectivity. Proceedings of the National Academy of Sciences, 106(6), 2035–2040. https://doi.org/10.1073/pnas.0811168106, 19188601 10.1073/pnas.0811168106 PMC2634800 19188601

[bib27] Hutchison, R. M., Womelsdorf, T., Gati, J. S., Everling, S., & Menon, R. S. (2013). Resting-state networks show dynamic functional connectivity in awake humans and anesthetized macaques. Human Brain Mapping, 34(9), 2154–2177. https://doi.org/10.1002/hbm.22058, 22438275 22438275 10.1002/hbm.22058 PMC6870538

[bib28] Iacovella, V., Faes, L., & Hasson, U. (2018). Task-induced deactivation in diverse brain systems correlates with interindividual differences in distinct autonomic indices. Neuropsychologia, 113, 29–42. https://doi.org/10.1016/j.neuropsychologia.2018.03.005, 29530799 29530799 10.1016/j.neuropsychologia.2018.03.005

[bib29] Iacovella, V., & Hasson, U. (2011). The relationship between bold signal and autonomic nervous system functions: Implications for processing of “physiological noise.” Magnetic Resonance Imaging, 29(10), 1338–1345. https://doi.org/10.1016/j.mri.2011.03.006, 21543181 21543181 10.1016/j.mri.2011.03.006

[bib30] Jenkinson, M., Beckmann, C. F., Behrens, T. E., Woolrich, M. W., & Smith, S. M. (2012). FSL. Neuroimage, 62(2), 782–790. https://doi.org/10.1016/j.neuroimage.2011.09.015, 21979382 21979382 10.1016/j.neuroimage.2011.09.015

[bib31] Kelly, A. C., Uddin, L. Q., Biswal, B. B., Castellanos, F. X., & Milham, M. P. (2008). Competition between functional brain networks mediates behavioral variability. NeuroImage, 39(1), 527–537. https://doi.org/10.1016/j.neuroimage.2007.08.008, 17919929 17919929 10.1016/j.neuroimage.2007.08.008

[bib32] Kim, J., Criaud, M., Cho, S. S., Díez-Cirarda, M., Mihaescu, A., Coakeley, S., … Strafella, A. P. (2017). Abnormal intrinsic brain functional network dynamics in Parkinson’s disease. Brain, 140(11), 2955–2967. https://doi.org/10.1093/brain/awx233, 29053835 29053835 10.1093/brain/awx233 PMC5841202

[bib33] Koba, C. (2021). NetNeuroSleepyBrain, GitHub, https://github.com/KobaCemal/SleepyBrain

[bib34] Law, I. (1998). Parieto-occipital cortex activation during self-generated eye movements in the dark. Brain, 121(11), 2189–2200. https://doi.org/10.1093/brain/121.11.2189, 9827777 9827777 10.1093/brain/121.11.2189

[bib35] Liu, T. T., Nalci, A., & Falahpour, M. (2017). The global signal in fMRI: Nuisance or information? NeuroImage, 150, 213–229. https://doi.org/10.1016/j.neuroimage.2017.02.036, 28213118 28213118 10.1016/j.neuroimage.2017.02.036 PMC5406229

[bib36] Marx, E., Stephan, T., Nolte, A., Deutschländer, A., Seelos, K. C., Dieterich, M., & Brandt, T. (2003). Eye closure in darkness animates sensory systems. NeuroImage, 19(3), 924–934. https://doi.org/10.1016/S1053-8119(03)00150-2, 12880821 12880821 10.1016/s1053-8119(03)00150-2

[bib37] McAvoy, M., Larson-Prior, L., Ludwikow, M., Zhang, D., Snyder, A. Z., Gusnard, D. L., … d’Avossa, G. (2012). Dissociated mean and functional connectivity bold signals in visual cortex during eyes closed and fixation. Journal of Neurophysiology, 108(9), 2363–2372. https://doi.org/10.1152/jn.00900.2011, 22875902 22875902 10.1152/jn.00900.2011 PMC3545171

[bib38] Mišic´, B., Betzel, R. F., De Reus, M. A., Van Den Heuvel, M. P., Berman, M. G., McIntosh, A. R., & Sporns, O. (2016). Network-level structure-function relationships in human neocortex. Cerebral Cortex, 26(7), 3285–3296. https://doi.org/10.1093/cercor/bhw089, 27102654 27102654 10.1093/cercor/bhw089 PMC4898678

[bib39] Morita, K., Miura, K., Kasai, K., & Hashimoto, R. (2020). Eye movement characteristics in schizophrenia: A recent update with clinical implications. Neuropsychopharmacology Reports, 40(1), 2–9. https://doi.org/10.1002/npr2.12087, 31774633 31774633 10.1002/npr2.12087 PMC7292223

[bib40] Mort, D. J., Perry, R. J., Mannan, S. K., Hodgson, T. L., Anderson, E., Quest, R., … Kennard, C. (2003). Differential cortical activation during voluntary and reflexive saccades in man. NeuroImage, 18(2), 231–246. https://doi.org/10.1016/S1053-8119(02)00028-9, 12595178 12595178 10.1016/s1053-8119(02)00028-9

[bib41] Nickerson, L. D., Smith, S. M., Öngür, D., & Beckmann, C. F. (2017). Using dual regression to investigate network shape and amplitude in functional connectivity analyses. Frontiers in Neuroscience. https://doi.org/10.3389/fnins.2017.00115, 28348512 10.3389/fnins.2017.00115 PMC5346569 28348512

[bib42] Nilsonne, G., Tamm, S., D’Onofrio, P., Thuné, H. Å., Schwarz, J., Lavebratt, C., … Åkerstedt, T. (2016). A multimodal brain imaging dataset on sleep deprivation in young and old humans. 1–27. Retrieved from https://openarchive.ki.se/xmlui/handle/10616/45181

[bib43] Nostro, A. D., Müller, V. I., Varikuti, D. P., Pläschke, R. N., Hoffstaedter, F., Langner, R., … Eickhoff, S. B. (2018). Predicting personality from network-based resting-state functional connectivity. Brain Structure and Function, 223(6), 2699–2719. https://doi.org/10.1007/s00429-018-1651-z, 29572625 29572625 10.1007/s00429-018-1651-z PMC5997535

[bib44] Nyström, M., & Holmqvist, K. (2010). An adaptive algorithm for fixation, saccade, and glissade detection in eyetracking data. Behavior Research Methods, 42(1), 188–204. https://doi.org/10.3758/BRM.42.1.188, 20160299 20160299 10.3758/BRM.42.1.188

[bib45] Parkes, L., Fulcher, B., Yücel, M., & Fornito, A. (2018). An evaluation of the efficacy, reliability, and sensitivity of motion correction strategies for resting-state functional MRI. NeuroImage, 171, 415–436. https://doi.org/10.1016/j.neuroimage.2017.12.073, 29278773 29278773 10.1016/j.neuroimage.2017.12.073

[bib46] Poldrack, R. A., Fletcher, P. C., Henson, R. N., Worsley, K. J., Brett, M., & Nichols, T. E. (2008). Guidelines for reporting an fMRI study. NeuroImage, 40(2), 409–414. https://doi.org/10.1016/j.neuroimage.2007.11.048, 18191585 18191585 10.1016/j.neuroimage.2007.11.048 PMC2287206

[bib47] Pretegiani, E., & Optican, L. M. (2017). Eye movements in Parkinson’s disease and inherited Parkinsonian syndromes. Frontiers in Neurology, 8, 592. https://doi.org/10.3389/fneur.2017.00592, 29170650 29170650 10.3389/fneur.2017.00592 PMC5684125

[bib48] Ramot, M., Wilf, M., Goldberg, H., Weiss, T., Deouell, L. Y., & Malach, R. (2011). Coupling between spontaneous (resting state) fMRI fluctuations and human oculo-motor activity. NeuroImage, 58, 213–225. https://doi.org/10.1016/j.neuroimage.2011.06.015, 21703354 21703354 10.1016/j.neuroimage.2011.06.015

[bib49] Rorden, C., Karnath, H. O., & Bonilha, L. (2007). Improving lesion-symptom mapping. Journal of Cognitive Neuroscience. https://doi.org/10.1162/jocn.2007.19.7.1081, 17583985 10.1162/jocn.2007.19.7.1081 17583985

[bib50] Rosenberg, M. D., Hsu, W.-T., Scheinost, D., Constable, T. R., & Chun, M. M. (2018). Connectome-based models predict separable components of attention in novel individuals. Journal of Cognitive Neuroscience, 30(2), 160–173. https://doi.org/10.1162/jocn_a_01197, 29040013 29040013 10.1162/jocn_a_01197

[bib51] Rubinov, M., & Sporns, O. (2010). Complex network measures of brain connectivity: Uses and interpretations. NeuroImage. https://doi.org/10.1016/j.neuroimage.2009.10.003, 19819337 10.1016/j.neuroimage.2009.10.003 19819337

[bib52] Rucci, M., & Poletti, M. (2015). Control and functions of fixational eye movements. Annual Review of Vision Science, 1, 499–518. https://doi.org/10.1146/annurev-vision-082114-035742, 27795997 10.1146/annurev-vision-082114-035742 PMC5082990 27795997

[bib53] Rudie, J. D., Brown, J., Beck-Pancer, D., Hernandez, L., Dennis, E., Thompson, P., … Dapretto, M. (2013). Altered functional and structural brain network organization in autism. NeuroImage: Clinical, 2, 79–94. https://doi.org/10.1016/j.nicl.2012.11.006, 24179761 10.1016/j.nicl.2012.11.006 PMC3777708 24179761

[bib54] Schaefer, A., Kong, R., Gordon, E. M., Laumann, T. O., Zuo, X.-N., Holmes, A. J., … Yeo, B. T. T. (2018). Local-global parcellation of the human cerebral cortex from intrinsic functional connectivity MRI. Cerebral Cortex. https://doi.org/10.1093/cercor/bhx179, 28981612 10.1093/cercor/bhx179 PMC6095216 28981612

[bib55] Shirer, W. R., Ryali, S., Rykhlevskaia, E., Menon, V., & Greicius, M. D. (2012). Decoding subject-driven cognitive states with whole-brain connectivity patterns. Cerebral Cortex. https://doi.org/10.1093/cercor/bhr099, 21616982 10.1093/cercor/bhr099 PMC3236795 21616982

[bib56] Siegel, S., & Castellan, N. J. (1956). Nonparametric statistics for the behavioral sciences (Vol. 7). New York: McGraw-Hill.

[bib57] Silberg, J. E., Agtzidis, I., Startsev, M., Fasshauer, T., Silling, K., Sprenger, A., … Lencer, R. (2019). Free visual exploration of natural movies in schizophrenia. European Archives of Psychiatry and Clinical Neuroscience, 269(4), 407–418. https://doi.org/10.1007/s00406-017-0863-1, 29305645 29305645 10.1007/s00406-017-0863-1

[bib58] Smith, S. M., Beckmann, C. F., Andersson, J., Auerbach, E. J., Bijsterbosch, J., Douaud, G., … WU-Minn HCP Consortium. (2013). Resting-state fMRI in the human connectome project. NeuroImage, 80, 144–168. https://doi.org/10.1016/j.neuroimage.2013.05.039, 23702415 23702415 10.1016/j.neuroimage.2013.05.039 PMC3720828

[bib59] Son, J., Ai, L., Lim, R., Xu, T., Colcombe, S., Franco, A. R., … Milham, M. (2019). Evaluating fMRI-based estimation of eye gaze during naturalistic viewing. Cerebral Cortex. https://doi.org/10.1093/cercor/bhz157, 31595961 10.1093/cercor/bhz157 PMC7132907 31595961

[bib60] Takarae, Y., Minshew, N. J., Luna, B., Krisky, C. M., & Sweeney, J. A. (2004). Pursuit eye movement deficits in autism. Brain, 127(12), 2584–2594. https://doi.org/10.1093/brain/awh307, 15509622 15509622 10.1093/brain/awh307

[bib61] West, G. L., Welsh, T. N., & Pratt, J. (2009). Saccadic trajectories receive online correction: Evidence for a feedback-based system of oculomotor control. Journal of Motor Behavior, 41(2), 117–127. https://doi.org/10.3200/JMBR.41.2.117-127, 19201682 19201682 10.3200/JMBR.41.2.117-127

[bib62] Xu, P., Huang, R., Wang, J., Van Dam, N. T., Xie, T., Dong, Z., … Luo, Y.-J. (2014). Different topological organization of human brain functional networks with eyes open versus eyes closed. NeuroImage, 90, 246–255. https://doi.org/10.1016/j.neuroimage.2013.12.060, 24434242 24434242 10.1016/j.neuroimage.2013.12.060

[bib63] Yarkoni, T., Poldrack, R. A., Nichols, T. E., Van Essen, D. C., & Wager, T. D. (2011). Large-scale automated synthesis of human functional neuroimaging data. Nature Methods. https://doi.org/10.1038/nmeth.1635, 21706013 10.1038/nmeth.1635 PMC3146590 21706013

[bib64] Yellin, D., Berkovich-Ohana, A., & Malach, R. (2015). Coupling between pupil fluctuations and resting-state fMRI uncovers a slow build-up of antagonistic responses in the human cortex. NeuroImage, 106, 414–427. https://doi.org/10.1016/j.neuroimage.2014.11.034, 25463449 25463449 10.1016/j.neuroimage.2014.11.034

[bib65] Zacà, D., Hasson, U., Minati, L., & Jovicich, J. (2018). Method for retrospective estimation of natural head movement during structural MRI. Journal of Magnetic Resonance Imaging, 48(4), 927–937. https://doi.org/10.1002/jmri.25959, 29393987 29393987 10.1002/jmri.25959

[bib66] Zhang, Y., Yan, A., Liu, B., Wan, Y., Zhao, Y., Liu, Y., … Liu, Z. (2018). Oculomotor performances are associated with motor and non-motor symptoms in Parkinson’s disease. Frontiers in Neurology, 9, 960. https://doi.org/10.3389/fneur.2018.00960, 30546341 30546341 10.3389/fneur.2018.00960 PMC6280559

