# Supplementary material for: Spontaneous eye movements during eyes-open rest reduce resting-state-network modularity by increasing visual-sensorimotor connectivity
Source: Netw Neurosci. 2021 Jun 3;5(2):451–76. doi: 10.1162/netn_a_00186 (PMC8233114; doi:10.1162/netn_a_00186)
Supplement: Supplementary file 1 [file netn-05-451-s001.pdf]

Koba, C., Notaro, G., Tamm, S., Nilsonne, G. & Hasson, U. (2021). Supporting information for "Spontaneous eye movements during eyes-open rest reduce resting-state-network modularity by increasing visual-sensorimotor connectivity." *Network Neuroscience.* Advance publication. https://doi.org/10.1162/netn_a_00186

Journal: **NETWORK NEUROSCIENCE**

RESEARCH

Spontaneous eye-movements during eyes-open rest reduce resting-state-network modularity by increasing visual-sensorimotor connectivity

SUPPLEMENTARY TABLES

Regressor	Voxels	Location	CM x	CM y	CM y	Peak x	Peak y	Peak z
Blink	8	Left Superior Frontal Gyrus	-22.2	-5.6	74.6	-21.4	-3.4	73.5
Gaze X^2	54	Left Superior Frontal Gyrus	-16.6	-0.4	70.3	-14.6	-3.4	70.5

Table 1. Cluster coordinates of the regions identified by the eye tracking data.

	Time in relation to peak value in eye-tracking series (sec)								
	-10	-7.5	-5	-2.5	0	2.5	5	7.5	10
Velocity of vertical gaze location	0.146	0.245	0.313	0.338	0.328	0.280	0.205	0.117	0.031
Pupil size squared	0.101	0.093	0.078	0.060	0.073	0.088	0.101	0.109	0.108
Velocity of Gaze Amplitude	0.027	0.014	0.003	-0.015	-0.029	-0.033	-0.026	-0.007	0.020

Table 2. Numeric values describing kernels mediating the relationship between EO-EPI and eye tracking time series, for those eye tracking features for which the relation was statistically significant.

Voxels	Location	CM x	CM y	CM z	Peak x	Peak y	Peak z	Mean Delta	Mean Z	Effect Size
18517	Right Cingulate Gyrus	-4.1	21.4	40.0	-37.1	25.9	70.5	0.00012	3.41	0.37
5144	Right Lingual Gyrus	-2.0	68.6	7.5	10.1	66.4	-1.5	0.000151	3.23	0.35
2521	Left Thalamus	2.1	19.4	12.4	1.1	23.6	13.5	-0.000117	-3.29	0.36
1806	Left Cerebellum/Declive	4.3	79.7	-29.5	-16.9	84.4	-19.5	-0.00011	-3.039	0.33
668	Right Cerebellum/Inferior Semi-Lunar Lobule	-21.1	62.5	-52.5	-28.1	52.9	-52.5	0.00007	3.31	0.36
592	Left Inferior Parietal Lobule	45.1	55.7	38.3	57.4	59.6	43.5	-0.000094	-2.92	0.32
547	Middle Occipital Gyrus	-21.8	96.3	-0.9	-12.4	1.5	100.1	-0.00015	-3.15	0.35
488	Left Cerebellum/Inferior Semi-Lunar Lobule	21.7	62.8	-52.9	21.4	64.1	-52.5	0.000068	3.21	0.35

Table 3. Cluster mass and peak coordinates of the regions by EYE_{conv}.

Voxels	Location	CM x	CM y	CM y	Peak x	Peak y	Peak z	Mean Delta	Mean Z	Effect Size
2888	Cerebellar Vermis	0.1	27.9	-12.8	3.4	43.9	4.5	-0.01313	-3.12	0.34
1937	Right Postcentral Gyrus	-45.8	19.2	49.7	-39.4	21.4	70.5	0.01696	3.04	0.33
1901	Left Postcentral Gyrus	50.3	20.7	47	43.9	21.4	67.5	0.018062	3.07	0.34
631	Left Cuneus	0.6	79.9	23.4	-1.1	79.9	37.5	0.019491	2.90	0.32
517	Left Middle Occipital Gyrus	47.1	70.5	3.8	52.9	70.9	13.5	0.014119	2.80	0.31

Table 4. Cluster mass and peak coordinates of the regions that are identified by EYE_{raw}.

Location	Mean tSNR	SD of tSNR
Cerebellar Vermis	62.58	21.35
Right Postcentral Gyrus	68.74	29.89
Left Postcentral Gyrus	66.8	29.43
Left Cuneus	78.19	34.38
Left Middle Occipital Gyrus	78.01	32.49

Table 5. Mean tSNR values of the clusters identified by EYE_{raw}.

Location	Mean tSNR	SD of tSNR
Right Cingulate Gyrus	67.75	28.96
Right Lingual Gyrus	70.06	30.53
Left Thalamus	61.26	22.71
Left Cerebellum/Declive	45.87	25.49
Right Cerebellum/Inferior Semi-Lunar Lobule	48.76	25.30
Left Inferior Parietal Lobule	86.02	32.38
Middle Occipital Gyrus	45.10	25.21
Left Cerebellum/Inferior Semi-Lunar Lobule	44.55	25.29

Table 6. Mean tSNR values of the clusters identified by EYE_{conv}.

	**Sparsity=0.1**		**Sparsity=0.2**		**Sparsity=0.3**	
	**Mean raw**	**Mean clean**	**Mean raw**	**Mean clean**	**Mean raw**	**Mean clean**
Max Degree	146.03	144.23	220.88	219.23	278.88	279.02
Min Degree	0.36	0.35	3.863	3.963	13.113	13.363
Max Strength	91.07	88.84	119.66	117.13	135.16	132.57
Min Strength	0.18	0.17	1.457	1.524	3.934	4.033
Mean Strength	29.74	29.45	50.09	49.48	65.367	64.464
Max Cluster Coefficient	0.672	0.668	0.529	0.522	0.442	0.434
Min Cluster Coefficient	0.004	0.003	0.061	0.063	0.111	0.112
Mean Cluster Coefficient	0.339	0.336	0.308	0.303	0.283	0.278
Transitivity	0.359	0.352	0.323	0.315	0.296	0.289
Assortativity	0.4220	0.4207	0.354	0.349	0.305	0.298
Efficiency	0.262	0.261	0.287	0.285	0.292	0.289
Max Number of Community	5.57	5.55	3.938	3.963	3.475	3.500
Maximized Modularity	0.44	0.45	0.353	0.358	0.300	0.303
Max Betweenness Centrality	6979.50	6994.27	4529.85	4524.27	4086.70	4059.82
Min Betweenness Centrality	773.09	766.48	537.955	533.852	475.344	474.124

Table 7. Mean values of the network metrics derived from the Raw and Clean (EO-EPI-removed) functional connectivity matrices. Shown for the three largest

sparsity levels.

SUPPLEMENTARY FIGURES

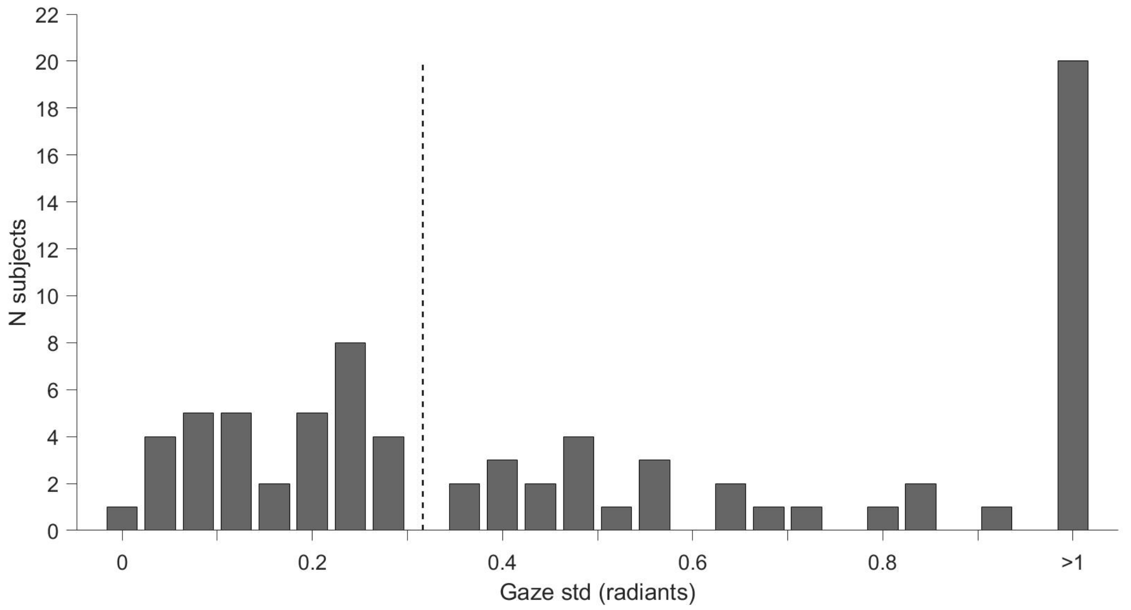

Figure 1. Histogram of the standard deviations of gaze norm value for all the 77 participants with available eye-tracking data. The vertical dotted line indicates the threshold value, $SD_{gaze} = 0.32$ used to discard datasets from 43 participants that contributed low quality eye-tracking data.

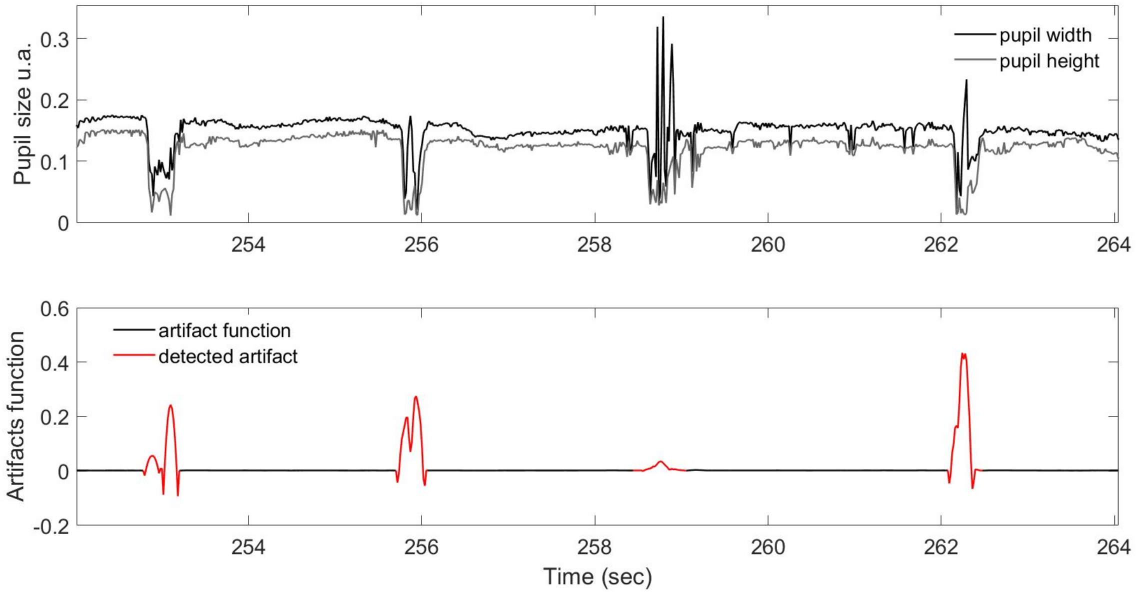

Figure 2. Example of how pupil data (height and width, upper panel) are transformed by the artifact function (lower panel). This transformation makes it possible to detect artifact peaks and duration.

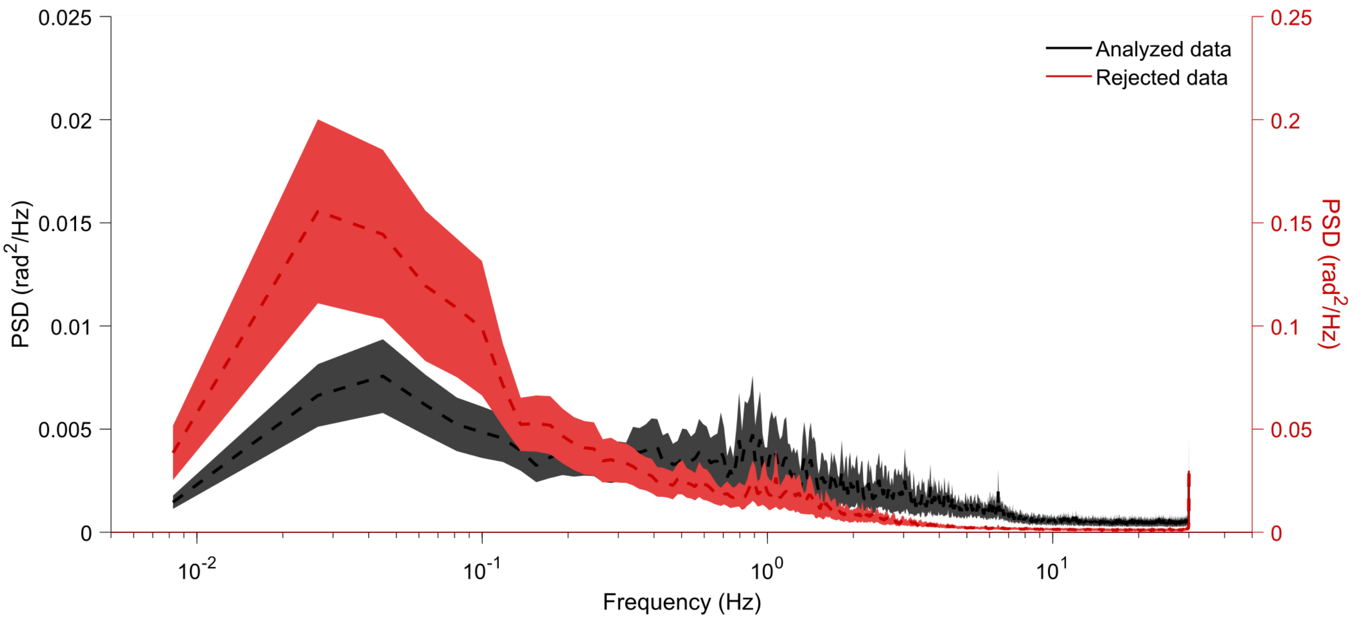

18 **Figure 3.** Power spectra of eye-tracking data for data rejected or maintained by the quality-control procedure. Note the dual Y-axis scales. Rejected data
19 presented power around one order of magnitude higher than maintained data. This difference was significant even for the highest measurable frequency, where
20 power in rejected data was 2.5 times higher than that maintained.

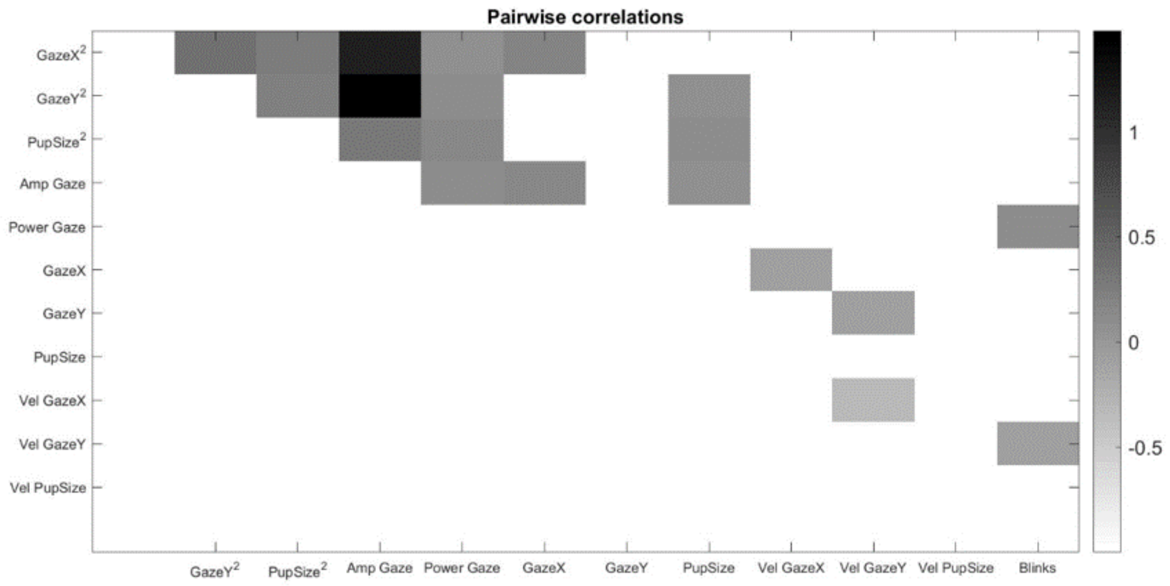

Figure 4. Correlation between different eye-tracking metrics. Only statistically significant correlations ar eshown. Correlation between Gaze Power and $Gaze^2$ or $Gaze^2$ reflects a spurious correlation as Gaze Power is the sum of those two terms.

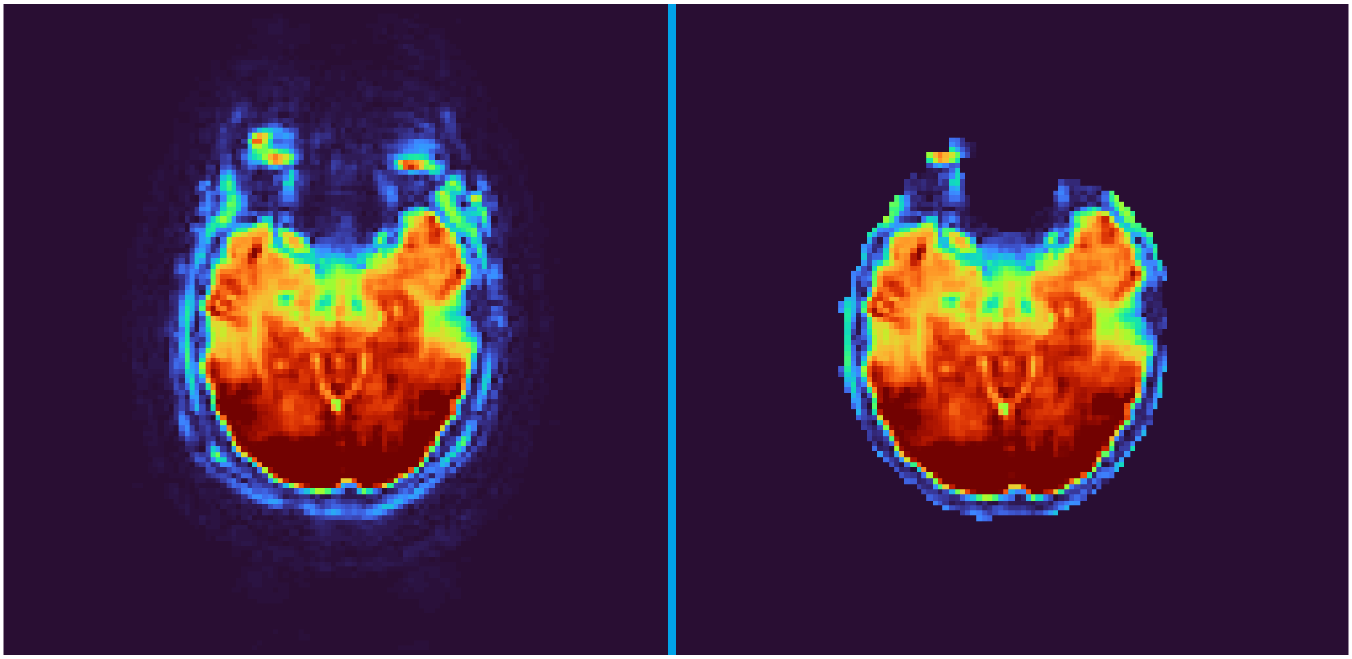

Figure 5. Impact of despiking on remaining signal in Eye Orbit area. Left image: without despiking. Right image: despiked. As shown, despiking removed a substantial amount of time series from the eye orbit area.

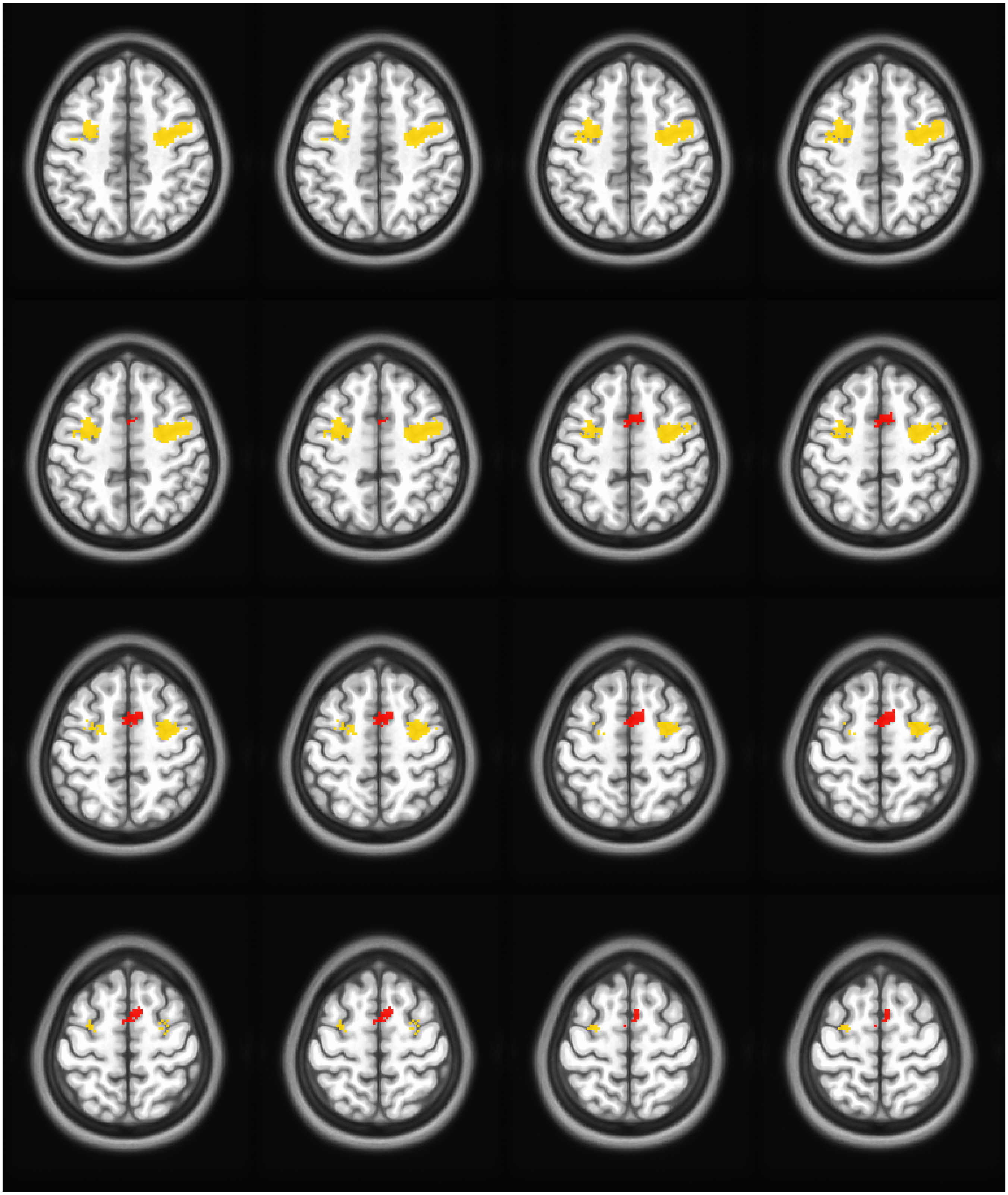

25 **Figure 6.** Anatomical regions of interest used in the analyses. Frontal eye fields (yellow) and Supplementary eye fields (red) ROIs derived from Neurosynth

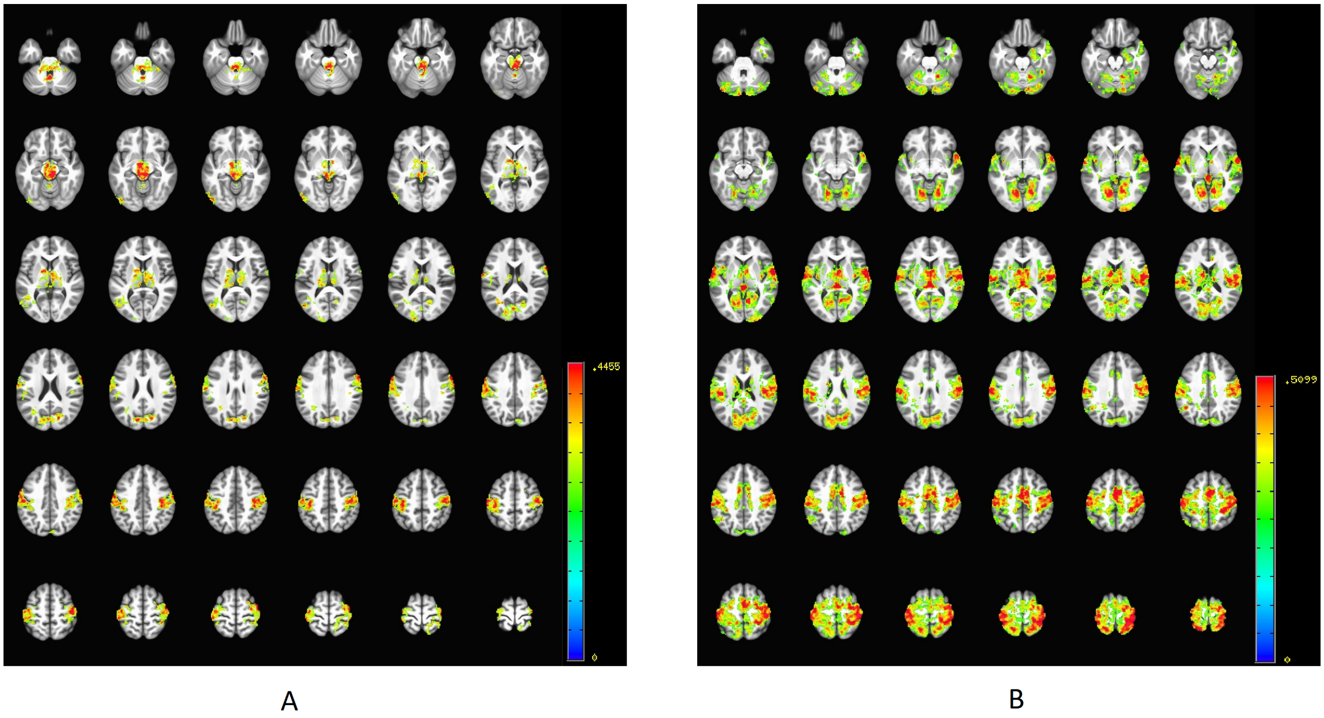

Figure 7. Effect sizes of EO-EPI/BOLD correlations computed for each voxel within statistically significant clusters. The panels show effect sizes for the non-convolved EO-EPI regressor (a) and for the convolved regressor (b).

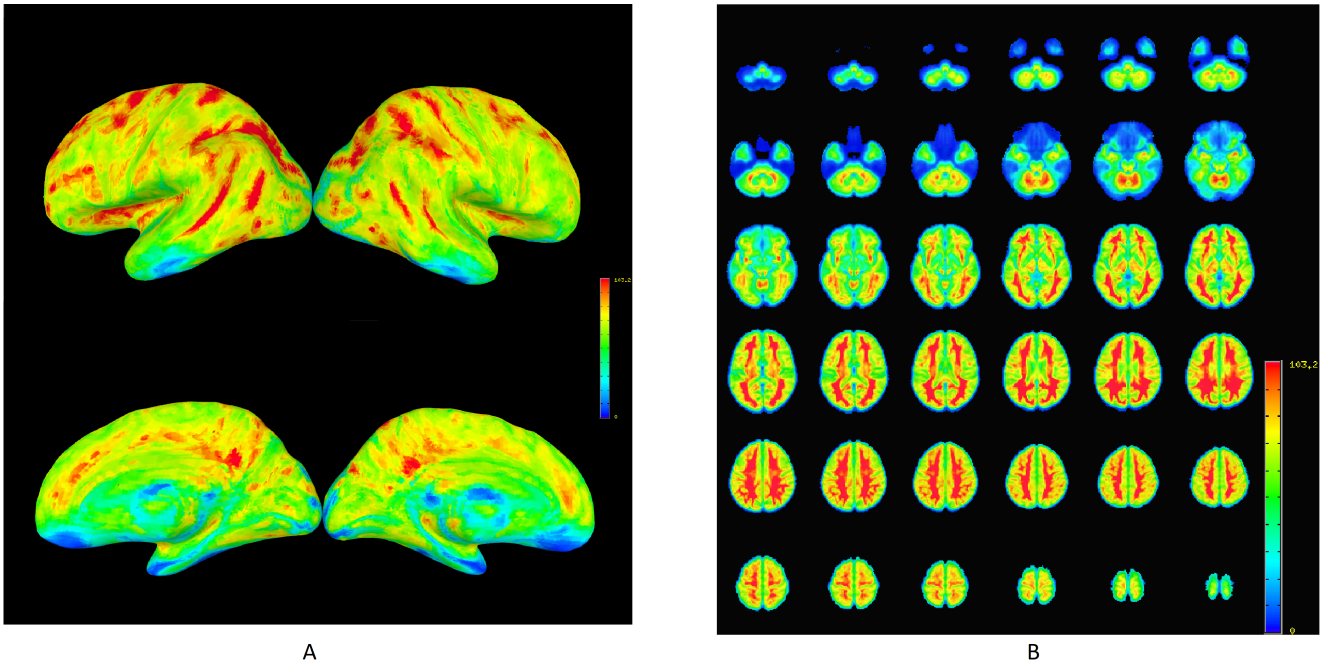

Figure 8. Temporal Signal-To-Noise (tSNR) values presented on cortical-surface (a) and axial-slices (b) representations. These were calculated as $Mean/SD$ of the raw time series prior to any pre-processing

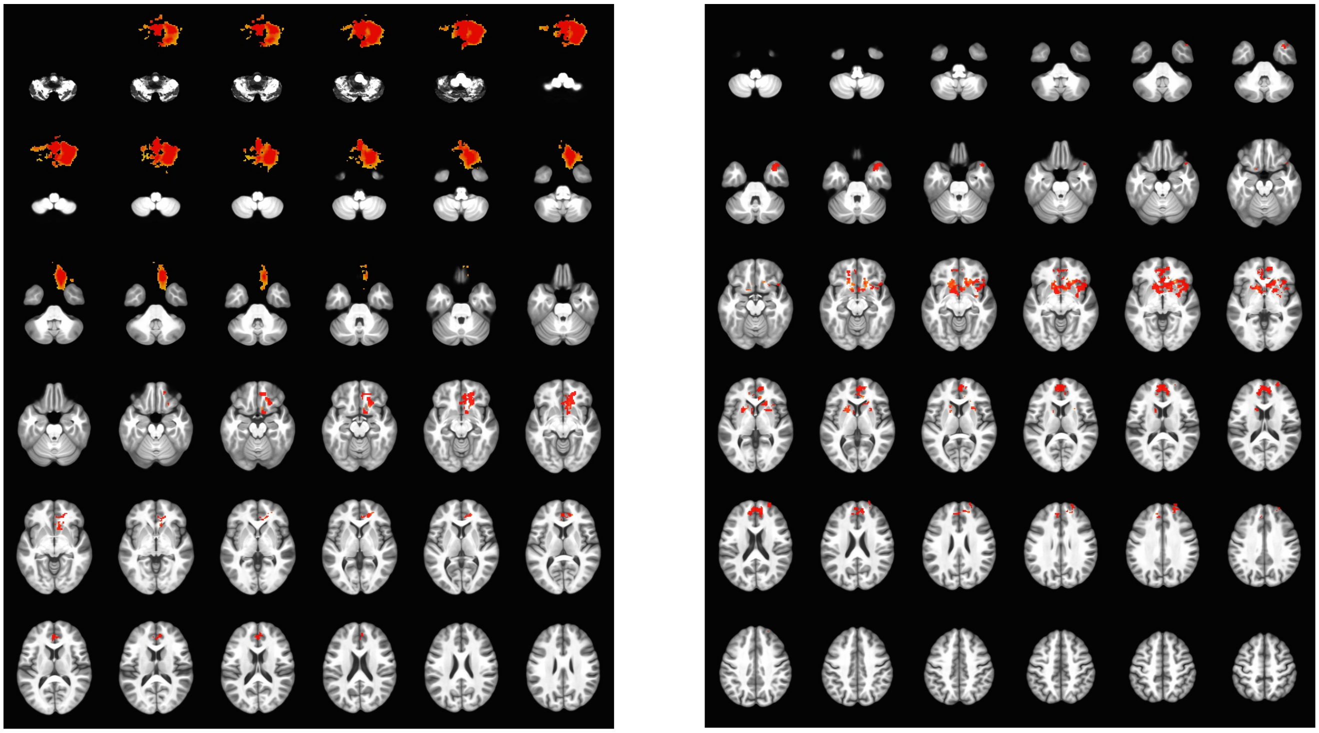

A B

Figure 9. Whole-brain connectivity for a control ROI defined in the maxillary sinus cavity below the eye. The panels show whole-brain connectivity for non-convolved regressor (a) and for the convolved regressor (b). Family-wise correction was implemented as for the EO-EPI regressor.

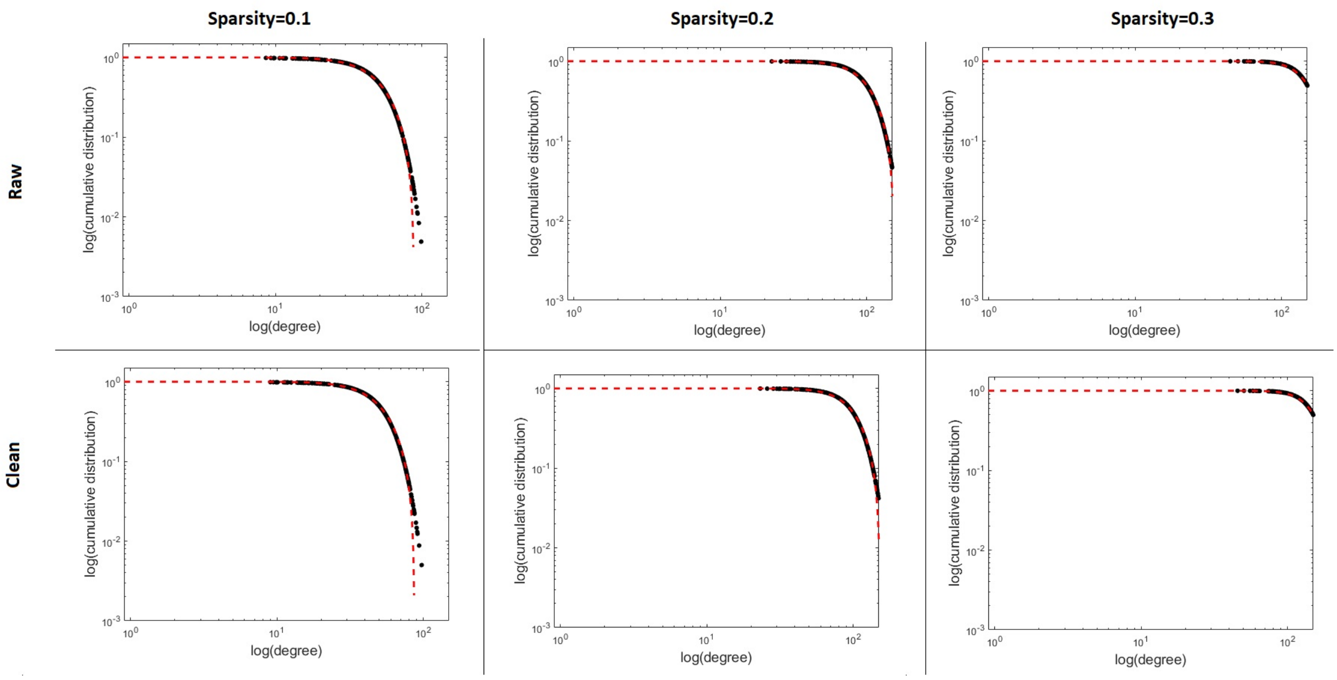

32 **Figure 10.** Mean degree distribution of raw and clean matrices across sparsity levels.

DESCRIPTION OF CHOSEN NETWORK METRICS

- **Degree**: Node degree is the number of links connected to the node. In directed networks, the in-degree is the number of inward links and the out-degree is the number of outward links. Connection weights are ignored in calculations.
- **Strength**: Node strength is the sum of weights of links connected to the node. In directed networks, the in-strength is the sum of inward link weights and the out-strength is the sum of outward link weights.
- **Clustering coefficient**: The clustering coefficient is the fraction of triangles around a node and is equivalent to the fraction of node's neighbors that are neighbors of each other.
- **Transitivity**: The transitivity is the ratio of triangles to triplets in the network and is an alternative to the clustering coefficient.
- **Assortativity**: The assortativity coefficient is a correlation coefficient between the degrees of all nodes on two opposite ends of a link. A positive assortativity coefficient indicates that nodes tend to link to other nodes with the same or similar degree.
- **Global Efficiency** : The global efficiency is the average inverse shortest path length in the network, and is inversely related to the characteristic path length.
- **Number of communities**: The optimal community structure is a subdivision of the network into nonoverlapping groups of nodes in a way that maximizes the number of within-group edges, and minimizes the number of between-group edges. To calculate maximum number of community for each network, we recorded the largest number of community.
- **Modularity**: The modularity is a statistic that quantifies the degree to which the network may be subdivided into such clearly delineated groups.
- **Betweenness Centrality**: Node betweenness centrality is the fraction of all shortest paths in the network that contains a given node. Nodes with high values of betweenness centrality participate in a large number of shortest paths
